# Quantum decoherence dynamics of divacancy spins in silicon carbide

**DOI:** 10.1038/ncomms12935

**Published:** 2016-09-29

**Authors:** Hosung Seo, Abram L. Falk, Paul V. Klimov, Kevin C. Miao, Giulia Galli, David D. Awschalom

**Affiliations:** 1 The Institute for Molecular Engineering, The University of Chicago, Chicago, Illinois 60615, USA; 2 IBM T.J. Watson Research Center, Yorktown Heights, New York 10598, USA; 3 Materials Science Division, Argonne National Laboratory, Lemont, Illinois 60439, USA

## Abstract

Long coherence times are key to the performance of quantum bits (qubits). Here, we experimentally and theoretically show that the Hahn-echo coherence time of electron spins associated with divacancy defects in 4*H*–SiC reaches 1.3 ms, one of the longest Hahn-echo coherence times of an electron spin in a naturally isotopic crystal. Using a first-principles microscopic quantum-bath model, we find that two factors determine the unusually robust coherence. First, in the presence of moderate magnetic fields (30 mT and above), the ^29^Si and ^13^C paramagnetic nuclear spin baths are decoupled. In addition, because SiC is a binary crystal, homo-nuclear spin pairs are both diluted and forbidden from forming strongly coupled, nearest-neighbour spin pairs. Longer neighbour distances result in fewer nuclear spin flip-flops, a less fluctuating intra-crystalline magnetic environment, and thus a longer coherence time. Our results point to polyatomic crystals as promising hosts for coherent qubits in the solid state.

## Abstract (web summary)

The length of time a qubit can store information is linked to its coherence time. Here, the authors demonstrate that industrially important crystals comprising more than one species can host qubits with unexpectedly long coherence times.

## Graphical abstract

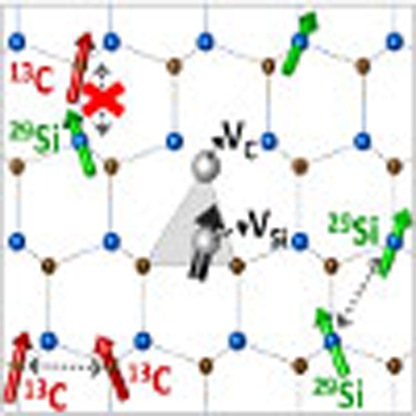

Impurity-based electron spins in crystals, such as the nitrogen vacancy (NV) centre in diamond^1^,^2^, donor spins in silicon^3^, transition-metal ions^4^ and rare-earth ions^5^ have recently attracted great interest as versatile solid-state quantum bits (qubits). Among the key measures for qubit performance, coherence times characterize the lifetime of a qubit. In quantum computing, long spin coherence times are necessary for executing quantum algorithms with many gates^6^. Qubits with robust coherence are also ideal systems for developing applications such as collective quantum memories^7^ and nano-scale quantum sensors^8^,^9^. Nonetheless, interactions between the spin qubit and the bath of paramagnetic nuclei in the crystal eventually limit the qubit's coherence^10^,^11^,^12^. One of the standard measures of spin coherence time is the ensemble Hahn-echo coherence time (*T*_2_)^13^. For NV centers in naturally isotopic diamond and for donor spins in natural silicon, *T*_2_ times have been measured to be 0.63 ms (ref. 14) and 0.5 to 0.8 ms (refs 15, 16, 17), respectively. These are set by the presence of naturally occurring ^13^C (1.1%, *I*_C_=1/2) isotopes^11^,^12^,^18^,^19^,^20^,^21^,^22^ and ^29^Si (4.7%, *I*_Si_=1/2) isotopes^10^,^23^,^24^,^25^. For Mn:ZnO, a 0.8-ms *T*_2_ time has been reported^4^, which is set by the ^67^Zn (4.1%, *I*_Zn_=5/2) isotopic concentration.

Several techniques can be used to extend spin coherence, including isotopic purification^12^,^25^, dynamical decoupling^26^,^27^,^28^ and the use of particular ‘clock transitions' that are immune to external magnetic perturbations^29^,^30^,^31^. These techniques cannot be used in all applications, however, and moreover, the extent to which spin coherence can be extended is typically correlated to the original *T*_2_ time. Therefore, the Hahn-echo *T*_2_ time in a naturally isotopic crystal remains an important metric for qubit performance.

Recently, Christle *et al*.^32^ reported a *T*_2_ time of 1.2 ms for divacancies in SiC, which are spin-1 defects^33^,^34^,^35^,^36^,^37^,^38^,^39^,^40^,^41^,^42^. However, the spin dynamics underlying this coherence time were not understood. Naturally isotopic SiC contains both ^29^Si (4.7%) and ^13^C (1.1%) isotopes. Nevertheless, in spite of having a higher nuclear spin density than natural diamond, SiC was able to host qubits with a much longer *T*_2_ time than those of NV centers, implying a suppression of nuclear spin bath fluctuations. Yang *et al*.^43^ recently published an insightful theoretical paper on the nuclear-bath driven decoherence of single-silicon vacancy (V_Si_) in SiC, a spin-3/2 defect^44^,^45^,^46^,^47^,^48^,^49^,^50^. Using the cluster-correlation expansion (CCE) theory^51^, they showed that heterogeneous nuclear spin flip-flop processes are suppressed in SiC due to the difference between the gyromagnetic ratios of ^29^Si and ^13^C nuclear spins (or heterogeneity). Similar heterogeneity and bath decoupling effects were also discussed for GaAs quantum dots^52^. Based on the bath decoupling effect, Yang *et al*.^43^, suggested that the spin coherence time in naturally isotopic SiC would be longer than that of the NV centre in diamond. However, direct experimental verification in SiC has been challenging using single V_Si_ spins^48^,^53^, partly because hyperfine coupling to the *S*=3/2 state gives rise to irregular coherence patterns^43^.

Here, we combine experiment and theory to study the decoherence dynamics of the *S*=1 electronic spin ensemble of the neutral (*kk*)-divacancy in 4*H*–SiC over a wide range of magnetic fields. We use optically detected magnetic resonance (ODMR)^36^ and a first-principles microscopic quantum-bath model^54^ combined with the CCE method^51^,^52^ to demonstrate that the *T*_2_ time of the divacancy spin in 4*H*–SiC can reach 1.3 ms, an unusually long *T*_2_ time. Our theoretical results successfully explain all the important features found in our experiment such as the behaviour of *T*_2_ as a function of magnetic field and the fine details in the electron spin echo envelop modulations (ESEEM)^13^. In particular, by studying ensembles of *S*=1 centers instead of single *S*=3/2 centers, we provide strong evidence that in SiC, the Si and C nuclear spin baths are decoupled at moderate magnetic field (∼30 mT), confirming the predictions of Yang *et al*.^43^. In addition to verifying Yang's predictions, we show that a key factor underlying the long coherence times in SiC is the fact that homo-nuclear spin pairs in this binary crystal must be at least two lattice sites away from each other. This separation limits the strength, and therefore the flip-flop rate, of the most strongly coupled spin pairs.

## Results

### Optically detected spin coherence in SiC

**Figure 1:**
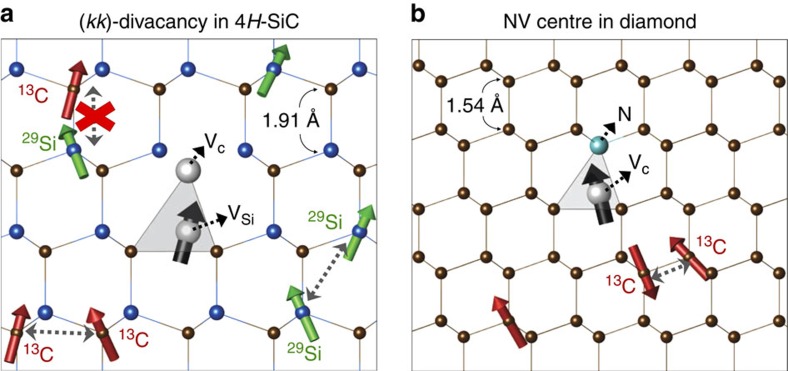
Defect spin qubits in nuclear spin baths. (**a**) A depiction of the neutral (*kk*)-divacancy defect complex in 4*H*–SiC, in which a carbon vacancy (V_C_, white sphere) at a quasi-cubic site (*k*) is paired with a silicon vacancy (V_Si_, white sphere) formed at the nearest neighbouring (*k*) site. (**b**) A depiction of the negatively charged NV centre in diamond, which consists of a carbon vacancy (V_C_, white sphere) paired with a substitutional nitrogen impurity (N, green sphere). Both defects have the same C_3v_ symmetry (denoted by a grey pyramid) and spin-1 (black arrow) triplet ground state mainly derived from the surrounding carbon sp^3^ dangling bonds. While the NV center spin is coupled to a homogeneous ^13^C nuclear spin bath (1.1%, *I*_C_=1/2 represented with red arrows), the divacancy spin interacts with a heterogeneous nuclear spin bath of ^13^C and ^29^Si (4.7%, *I*_Si_=1/2 represented with green arrows).

Our experiments use 4*H*–SiC wafers (purchased from Cree, Inc.) with vacancy complexes intentionally incorporated during crystal growth. The divacancy density is ∼10^12^ cm^−3^ (ref. 37). In this study, we consider the (*kk*)-divacancy^36^,^37^, which is schematically shown in Fig. 1. We use a 975 nm laser diode to illuminate the sample, which, through ODMR, polarizes the electronic ground state of the divacancies into their *m*_s_=0 state^36^,^37^. The divacancies exhibit more intense photoluminescence (PL) in their *m*_s_=±1 state^36^,^37^ than in their *m*_s_=0 state, allowing the spin of the defects to be read out via the PL intensity. We use a movable permanent magnet to apply a *c-*axis-oriented magnetic field (*B*)^36^. To measure the pure spin dephasing rate, we perform standard Hahn-echo pulse sequence (*π*/2 pulse−*t*_free_/2−*π* pulse−*t*_free_/2−*π*/2 pulse)^13^ measurements. The first *π*/2 pulse creates a superposition of the *m*_s_=+1 and *m*_s_=0 states, and the following *π* pulse reverts the spin precession after the *t*_free_/2 free evolution. At the end of the Hahn-echo sequence, the spin coherence is refocused, removing the effects of static magnetic inhomogeneity. The last *π*/2 pulse converts the phase difference in the superposition state to a population difference in the *m*_s_=+1 and *m*_s_=0 states, which we then measure through a change in the PL intensity.

**Figure 2:**
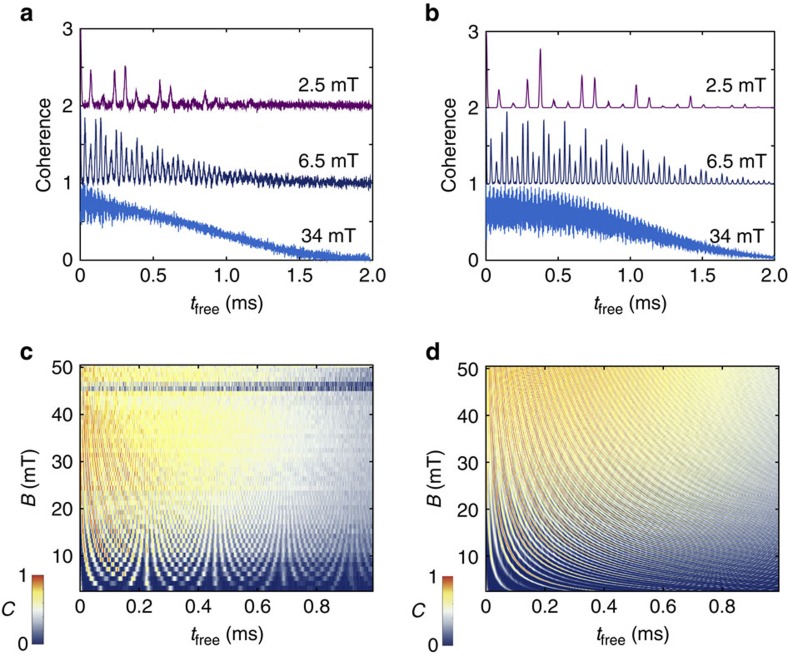
Hahn-echo coherence of the divacancy ensemble in 4*H*–SiC. (**a**,**b**) Experimental (**a**) and theoretical (**b**) Hahn-echo coherence of the *m*_s_=+1 to *m*_s_=0 ground-state spin transition of the divacancy ensemble with the *c*-axis-oriented magnetic field (*B*) at three different values. The experimental data was taken at *T*=20 K. (**c**,**d**) Experimental (**c**) and theoretical (**d**) Hahn-echo coherence of the spin transition from **a** and **b**, respectively, as a continuous function of free evolution time (*t*_free_) and *B*. The early loss of coherence near 47 mT in **c** corresponds to the spin triplet's ground-state level anti-crossing (GSLAC).

**Figure 3:**
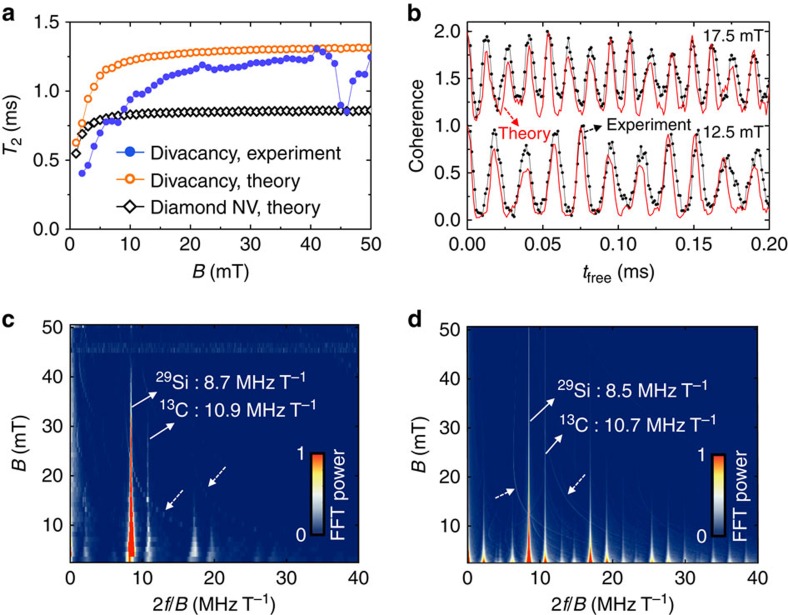
Analysis of the divacancy coherence. (**a**) Experimental Hahn-echo coherence time (*T*_2_) of the divacancy spin ensemble as a function of magnetic field (*B*) (filled circles) compared with theoretical *T*_2_ of the divacancy (empty circles) and theoretical *T*_2_ of the NV centre in diamond (empty diamonds). The divacancy *T*_2_ rises significantly, up to ∼20 mT, and is then roughly constant, except for a dip at 47 mT, corresponding to the ground-state level anti-crossing (GSLAC). (**b**) A direct comparison between the theoretical (red curve) and experimental (black curve) Hahn-echo coherence of the divacancy spin ensemble at two different magnetic fields of 17.5 mT (up) and 12.5 mT (down). (**c**,**d**) Experimental (**c**) and theoretical (**d**) FFT power spectrum of the *m*_s_=+1 to *m*_s_=0 ground-state spin coherence data of the divacancy from Fig. 2c,d, respectively. The frequency axis (*x* axis) is normalized to *B*, so that the nuclear precession frequencies appear as vertical lines. Harmonics of these frequencies can also be seen both in theory and experiment. After 7 mT, the FFT intensities diminish as *B* is increased. The hyperbolic features denoted by dotted arrows correspond to weak hyperfine interactions.

In Fig. 2, we show the measured Hahn-echo coherence of the divacancy ensemble at three representative magnetic fields and as a continuous function of magnetic field. At low magnetic fields, for example, 2.5 and 6.5 mT shown in Fig. 2a, the spin coherence rapidly collapses and revives as a function of time. Simultaneously, its envelop decays over time, leading to the loss of coherent phase information within 1 ms. In Fig. 2, we observe that this spin decoherence is largely suppressed and that the coherence is further extended as the static magnetic field is increased. We show the *T*_2_ as a function of magnetic field in Fig. 3a. We find that *T*_2_ increases as a function of magnetic field and saturates to 1.3 ms at a magnetic field of roughly 30 mT. There is a dip in *T*_2_ at a magnetic field of ∼47 mT, which is also visible in Fig. 2c as a coherence drop. This magnetic field converts to 1.31 GHz energy splitting, corresponding to the zero-field splitting of the (*kk*)-divacancy^37^. The coherence drops at this ground-state level anti-crossing as the *m*_s_=0 spin state can significantly mixes with *m*_s_=−1 spin sublevel.

### Quantum bath approach to decoherence

To understand the decoherence dynamics observed in experiment, we use quantum-bath theory, which describes the qubit decoherence occurring due to the entanglement between the qubit and the environment^54^. We apply the same theory to the NV centre and to the (*kk*)-divacancy spin so as to compare results consistently and to understand the underlying physical reasons responsible for their difference. The two defects share many common features^34^,^35^,^36^,^39^. For example, the *c-*axis-oriented (*kk*) divacancy (Fig. 1a) exhibits the same C_3v_ point-group symmetry and ^3^A_2_ spin triplet ground state as the NV centre in diamond (Fig. 1b). Furthermore, similar to the NV centre, the divacancy ground state is mainly derived from the three carbon sp^3^ orbitals localized around the silicon vacancy site in SiC. The only difference between the divacancy-in-SiC model and the NV-centre-in-diamond model is the type of nuclear spin bath along with their lattice structures as shown in Fig. 1a,b, respectively. We note that the dynamics of NV-centre decoherence has been well-understood, and that our results are in excellent agreement with those previously reported in the literature^18^,^19^,^22^. In our model, we ignore any possible effects arising from the nuclear and electronic spin-lattice relaxation. (See Supplementary Note 1 for further discussions). To solve the central spin model, we use the CCE method^51^,^52^, and we systematically approximate the coherence function at different orders. No adjustable parameters are used. Further details on the theoretical methods and the numerical calculations can be found in the methods section and the Supplementary Notes 1–3, together with Supplementary Figs 1–8 and Supplementary Table 1.

In Fig. 2b,d, we show the theoretical Hahn-echo coherence functions of the divacancy spin, to be compared with the experimental coherence data shown in Fig. 2a,c, respectively: the agreement between theory and experiment is excellent. In Fig. 3a, we compare the theoretical *T*_2_ times of the divacancy to the experimentally measured *T*_2_ times. Both *T*_2_ curves rapidly increase as a function of the free evolution time (*t*_free_) up to a magnetic field of 20 mT. For *B*>30 mT, they both saturate at a limit of 1.3 ms, although the experimental *T*_2_ curve appears to saturate more slowly. The dip in *T*_2_ at a magnetic field around 47 mT is not found in the theory, because in our model, we did not consider spin mixing between *m*_s_=0 and *m*_s_=−1 near the ground-state level anti-crossing. As a verification of our methods, we also compare the computed and measured divacancy *T*_2_ times with the theoretical *T*_2_ times of the NV centre in diamond (Fig. 3a). The theoretical limit of the NV-centre *T*_2_ time is found to be ∼0.86 ms, in agreement with ensembles measurements^14^ and with previous theoretical results obtained by the disjoint-cluster method^18^ and an analytical method^22^. Our theoretical results confirm that the divacancy *T*_2_ time in naturally isotopic 4*H*–SiC is much longer than that of the NV centre in naturally isotopic diamond.

In Fig. 3b, we compare the theoretical and experimental coherence functions at two different magnetic fields (12.5 and 17.5 mT). We find that the measured oscillation pattern of the coherence is also well reproduced by the theory, including the relative peak height and width, further verifying our microscopic model comprising ^29^Si and ^13^C nuclear spins. In the presence of a static magnetic field, the ^29^Si and ^13^C nuclear spins precess at their respective Larmor frequencies and induce ESEEM^13^,^55^. In Fig. 3c,d, we compare the *B*-normalized fast Fourier transform (FFT) spectra of the full experimental and theoretical coherence functions shown in Fig. 2c,d, respectively. Two-peak structures are clearly seen, centered at the ^29^Si and ^13^C nuclear gyromagnetic ratios, which are 8.7 and 10.9 MHz T^−1^ in experiment, and 8.5 and 10.7 MHz T^−1^ in theory, respectively. In addition to the Larmor-frequency peaks, we observe faint, but appreciable hyperbolic features both in experiment and theory as denoted by dotted arrows in Fig. 3c,d, respectively.

Since the ESEEM spectrum is derived from the independent precession of nuclear spins, the generic features of the spectrum may be understood using the analytical solution of an independent nuclear spin model (see Supplementary Fig. 5)^13^,^55^:


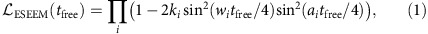


where *i* labels individual ^29^Si and ^13^C nuclear spins in the nuclear spin bath, *k*_*i*_ is a modulation depth parameter, *w*_*i*_ is the frequency of the *i*th nuclear spin and *a*_*i*_ is a frequency that depends on the hyperfine coupling parameters and the nuclear frequency (Supplementary Note 3). When the electron spin is in the *m*_*s*_=0 state, the hyperfine field on the nuclear spins is zero, leading to coherence oscillations at the bare nuclear frequencies. For the electron spin in the *m*_s_=+1 state, each nuclear spin experiences a different hyperfine field depending on its position relative to the electron spin, giving rise to the hyperfine-frequency term (*a*_*i*_) in equation (1). We note that these *a*_*i*_terms in equation (1) due to weak hyperfine interactions give rise to the hyperbolic features found in the FFT spectra shown in Fig. 3c,d. We find similar hyperbolic features in the computed FFT spectrum of the NV centre in diamond (not shown), although less pronounced compared with that of the SiC divacancy FFT spectrum. The modulation depth parameter, *k*_*i*_ in equation (1) is inversely proportional to the magnetic field (Supplementary Note 3), explaining the suppression of the oscillation amplitude at a large magnetic field found both in experiment and theory, as shown in Fig. 2a,b, respectively. The FFT intensities also diminish as *B* is increased for the same reason as shown in Fig. 3c,d.

### Suppressed qubit decoherence in silicon carbide

We now turn our attention to the microscopic origin of the longer *T*_2_ time of the divacancy (1.3 ms at *B*=30 mT) compared with that of the NV centre (0.8 ms at *B*=30 mT), in spite of the much larger number of nuclear spins in the SiC lattice. By comparing calculations performed at different CCE orders (Supplementary Fig. 3), we find that for both NV and the divacancy the computed Hahn-echo coherence time is numerically converged at the CCE-2 level of theory. This finding indicates that the dominant contribution to decoherence comes from pairwise nuclear transitions induced by nuclear dipole–dipole couplings. The decoherence of the NV centre in diamond is mainly caused by pairwise nuclear spin flip-flop transitions (↑↓↔↓↑), which induce magnetic noise at the NV centre through the hyperfine interaction. Other pairwise nuclear spin transitions, such as co-flips (↑↓↔↓↑), are suppressed at magnetic fields larger than roughly 10 mT. These results agree well with those previously reported for NV centers in diamond^18^,^19^,^22^.

In 4*H*–SiC, the nuclear spin interactions can be grouped in two categories: heterogeneous, between ^13^C and ^29^Si, and homogeneous interactions between nuclear spins of the same kind. The Hahn-echo coherence function of the divacancy can then be written as:


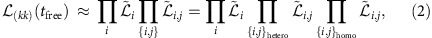


where 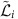 is a single-correlation term from the *i*th nuclear spin and 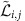 is an irreducible pair-correlation contribution from the *i−j* nuclear spin pair. The product over {*i,j*}_hetero_ include all ^13^C–^29^Si nuclear spin interactions, while the product over {*i,j*}_homo_ include all ^13^C–^13^C and ^29^Si–^29^Si spin pairs. We define the following heterogeneous and homogeneous coherence functions:


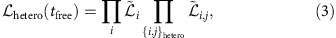



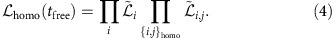


**Figure 4:**
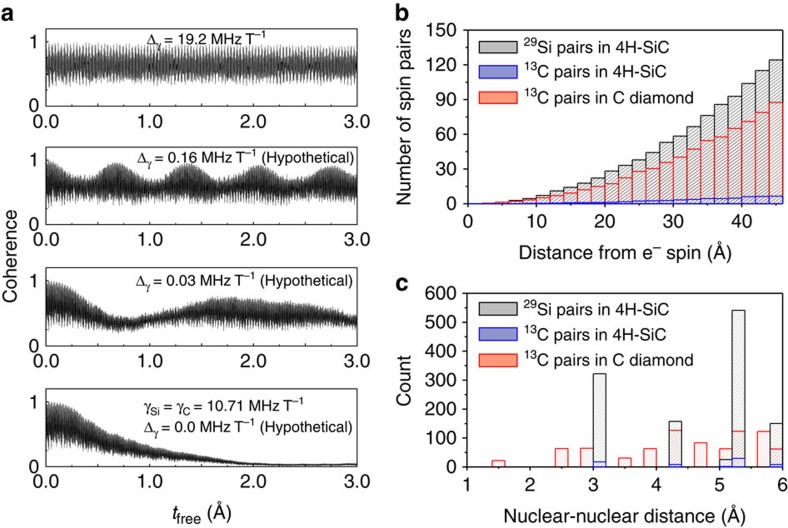
Effective decoupling of the ^13^C and ^29^Si spin baths in 4*H*–SiC. (**a**) The theoretical Hahn-echo coherence function of the divacancy ensemble at *B*=30 mT, calculated by only including the single- and heterogeneous pair-correlation contributions as defined in equation (3) and by varying the gyromagnetic ratio of ^29^Si (*γ*_Si_) as a theoretical parameter while that of ^13^C (*γ*_C_) is fixed at its experimental value. (**b**) The average number of homogeneous nuclear spin pairs whose lengths are <6 Å, as a function of distance from the divacancy qubit in 4*H*–SiC and from the NV centre in diamond. The centre-of-mass of a nuclear spin pair is used to measure the distance from the qubit. (**c**) The spatial distribution of homogeneous nuclear spin pairs in 4*H*–SiC and in diamond. The shortest homogeneous nuclear spin pair in diamond is 1.54 Å, corresponding to the C–C bond length, while that of the homogeneous nuclear spin pair in 4*H*–SiC is 3.07 Å, which is the second nearest neighbouring Si–Si or C–C distances.

To investigate the effect of the heterogeneity, we vary the gyromagnetic ratio of ^29^Si (*γ*_Si_) as a theoretical parameter while that of ^13^C (*γ*_C_) is fixed at the experimental value. In Fig. 4, 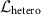 is shown at four different *γ*_Si_ values at a magnetic field of 30 mT. We find that there would be a significant decay of 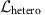 if the ^29^Si and ^13^C gyromagnetic ratios were hypothetically the same (*Δ*_*γ*_ ≡ *γ*_C_−*γ*_Si_=0), while small differences in the gyromagnetic ratios (*Δ*_*γ*_=0.03 MHz T^−1^ and 0.16 MHz T^−1^ for the two middle plots in Fig. 4a) are sufficient to significantly suppress the decay. Furthermore, when using the experimental values of *γ*_Si_ and *γ*_C_, 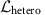 does not show any envelop decay, indicating no contribution from pairwise heterogeneous nuclear spin transitions for *B*>10 mT. Due to the sign difference between the gyromagnetic ratios of ^29^Si and ^13^C (*γ*_Si_<0, *γ*_C_>0), when *B*>10 mT, the lowest-energy ^29^Si - ^13^C pairwise spin transition is the co-flip of the nuclear spins (↑↓↔↓↑). In addition to the hyperfine field difference on the order of few kHz, the difference between *γ*_Si_ and *γ*_C_ gives an extra Zeeman contribution to the energy gap (∼0.2 MHz at *B*=10 mT) for the co-flips, which is larger than the typical heterogeneous dipole–dipole transition rate (∼kHz) in 4*H*–SiC.

The absence of heterogeneous nuclear spin transitions amounts to a decoupling of the nuclear spin bath in SiC and therefore the Hahn-echo coherence function is given by:


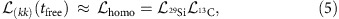


where 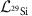 and 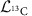 are the Hahn-echo coherence functions of the divacancy spin coupled to ^29^Si nuclear spins only and to ^13^C nuclear spins only, respectively. Since only transitions between homo-nuclear spins contribute to 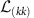, the density of nuclear spins contributing to the electron spin decoherence turns out to be similar to that found in diamond^53^, in spite of the total density of spins being much higher. However, this so-called dilution effect by itself would point to a similar electron spin decoherence rate in SiC and in diamond^53^, contrary to what is found experimentally (1.3-ms and 0.63-ms *T*_2_ time in SiC and diamond, respectively).

To better understand the nature of the nuclear spin baths in SiC, we compare in Fig. 4b the ensemble-averaged numbers of homogeneous nuclear spin pairs that are contributing to the decoherence of the divacancy in 4*H*–SiC and of the NV centre in diamond. In the former case, the homogeneous ^29^Si (4.7%) spin pairs are the dominant source of the qubit decoherence, and their number is larger than that of the ^13^C (1.1%) spin pairs in diamond. However, being further apart, their contribution is weaker than that of the homo-nuclear spin pairs in diamond. In Fig. 4c the distributions of nuclear spin pairs shown in Fig. 4b, are reported as a function of nuclear–nuclear distance. In the case of the NV centre in diamond, there is a small but significant number of nuclear spin pairs at a distance <3.0 Å, including first-, second- and third nearest C–C neighbours. These spins exhibit strong secular dipole–dipole transition rates, ranging from 0.24 kHz to 2.06 kHz: while they are minority spin pairs in number, they account for more than 90% of the coherence decay for the NV centre in diamond (Supplementary Fig. 2e). In contrast, in 4*H*–SiC, the smallest distance between homogeneous spins is 3.1 Å, corresponding to the Si–Si or C–C neighbours in SiC. As a result, the secular dipole–dipole transition rates for all the homogeneous nuclear spin pairs in 4*H*–SiC turn out to be <0.08 kHz. Our results show that the absence of strongly coupled nuclear spin clusters in SiC plays a key role in explaining the surprisingly long divacancy *T*_2_ times.

### Isotopic purification to lengthen *T*_2_

**Figure 5:**
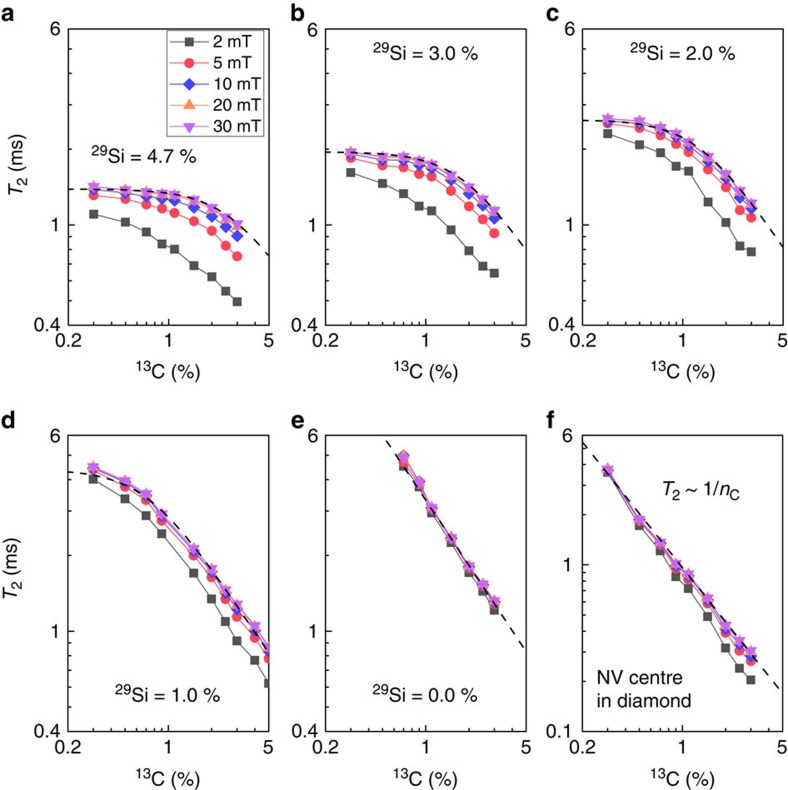
Divacancy coherence time in isotopically purified 4*H*–SiC. (**a**–**f**) Theoretical Hahn-echo coherence times (*T*_2_) of the divacancy ensemble in 4*H*–SiC (**a**–**e**) and the NV centre in diamond (**f**) as a function of ^13^C isotope concentration with a fixed ^29^Si concentration at 4.7% (**a**), 3.0% (**b**), 2.0% (**c**), 1.0% (**d**) and 0.0% (**e**) at five different magnetic fields. The black dashed line is the scaling law in equation (6) in the main text.

We showed that the coherence time of the divacancy in our naturally isotopic, semi-insulating 4*H*–SiC is 1.3 ms. In principle, the ^29^Si or ^13^C nuclei can be removed by isotopic purification, which is available in SiC (refs 56, 57), and a longer qubit coherence time could be achieved^12^,^18^,^24^,^58^. In Fig. 5, we report the Hahn-echo *T*_2_ of the divacancy ensemble in 4*H*–SiC computed as a function of the ^13^C concentration, while that of ^29^Si was fixed at given values, and we compare the results with those for the Hahn-echo *T*_2_ of the NV centre in diamond. In the case of the NV centre (Fig. 5f), we find that *T*_2_ scales as 1/*n*_c_ 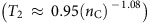, where *n*_c_ is the concentration of the ^13^C isotopes, in excellent agreement with previous theoretical^18^ and experimental^11^ findings.

In 4*H*–SiC, we observe that the divacancy *T*_2_ time increases as both ^29^Si and ^13^C concentrations are reduced. However, this increase does not appear to follow a simple power-law scaling behaviour. For example, in Fig. 5a, where the ^29^Si concentration is fixed at the experimental value of 4.7%, *T*_2_ is nearly constant as the ^13^C concentration is lowered below 1.1%. The behaviour of *T*_2_ is also significantly dependent on the applied magnetic field. We note that even if the ^13^C concentration is reduced, ^29^Si nuclear spins are still the majority ones, and thus responsible for limiting the coherence time. As the ^29^Si concentration is reduced from 4.7 to 0% (Fig. 5a–e, the behaviour of *T*_2_ as a function of ^13^C concentration becomes linear, similar to that of the NV centre in diamond. To rationalize the scaling behaviour of the divacancy *T*_2_, we compute the dependence of 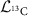 and 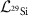 on the ^13^C and ^29^Si concentrations using equation (5), respectively, which we then fit with the compressed exponential decay function, 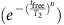. We find that *T*_2_ time of 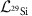 and 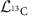 follows a simple scaling law as a function of nuclear spin concentration: 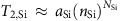 and 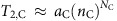 , with *a*_Si_=4.27 ms, *N*_Si_=−0.74, *a*_C_=3.31 ms and *N*_C_=−0.86, and the stretching exponent (*n*) is ∼2.6 for both C and Si when *B*>30 mT. This exponent is the same as that of the total coherence function, and although in good agreement with experiments (2.3), it is slightly larger. Using equation (5), we thus find that the divacancy *T*_2_ scales as follows:


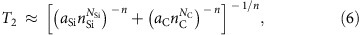


Equation (6), plotted as a dashed line in Fig. 5a–f, describes very accurately our full numerical simulation results at magnetic fields >20 mT. As noted above, however, the scaling behaviour significantly changes as the magnetic field is decreased under 20 mT and it cannot be described by equation (6). The inadequacy of equation (6) at low magnetic fields stems from the fact that heterogeneous nuclear spin transitions may occur, further limiting the *T*_2_ times. Therefore, the decoupling effect leading to equation (5) and thus, the scaling law in equation (6) are invalid at low magnetic fields.

## Discussion

We used a combined experimental and theoretical study to investigate the decoherence dynamics of divacancy spin qubits in 4*H*–SiC. We showed that, for *B*>30mT at *T*=20K, the *T*_2_ time of the divacancy reaches 1.3 ms almost two times longer than that of the NV centre. Using a combined microscopic quantum-bath model and a CCE computational technique, we found that 1.3 ms corresponds to the theoretical limit imposed by the presence of nuclear spins from naturally occurring ^29^Si and ^13^C isotopes. This limit is much longer than the corresponding one for the NV centre, which is ∼0.86 ms. The long spin coherence in SiC stems from the combination of two effects: the decoupling of the ^13^C and ^29^Si spin baths at a finite magnetic field, and the presence of active spins much further apart than those in diamond (for example, the closest ones belong to second neighbours in SiC and to first neighbours in diamond). We showed that, while the coherence of the NV centre is mainly limited by a few strongly interacting nuclear spin pairs belonging to nuclei within ∼3.0 Å of each other, in SiC, the homo-nuclear spin pair interactions are much weaker as they belong to second or further neighbours (see Fig. 1a). We note that the absence of strongly interacting nuclear spins in SiC is not a simple dilution effect. For example, the nuclear spin density in natural diamond is very low (1.1%), that is, it can be considered a diluted bath. Nevertheless, the distance between nuclei is such that strong nuclear spin interactions may arise, contributing to the decoherence of the NV centre in diamond. In SiC, Si and C spins have a much larger minimal distance from each other.

All experiments were performed at a low temperature (*T*=20 K) to exclude thermal effects and to focus on the pure dephasing of the divacancy spin (see Supplementary Note 1 for further discussions). Upon an increase of temperature, however, the divacancy *T*_2_ time would decrease significantly, as demonstrated in previous work^37^. In ref. 37, at low field, the *T*_2_ time of the divacancy spin was observed to decrease from 360 μs at 20 K to 50 μs at room temperature. In contrast, the NV-centre coherence has been known to be relatively insensitive to a temperature change, thus a long coherence time can be measured even at room temperature^14^. The insensitivity of the NV-centre coherence to temperature has been mainly attributed to the high Debye temperature and small spin–orbit coupling in diamond. However, the origin of the temperature dependence of the divacancy coherence in SiC is yet unknown.

Although overall, our theoretical and experimental results are in excellent agreement, we did find a few minor discrepancies. First, the ESEEM frequencies in experiment are blue-shifted by ∼0.2 MHz T^−1^ from the free ^13^C and ^29^Si frequencies. The blue-shift effect becomes prominent in the appearance of the coherence oscillation at a low magnetic field such as *B*=2.5 mT in Fig. 2a. When compared with the corresponding theoretical plot in Fig. 2b, the ESEEM peaks appear slightly faster in the experiment. Two possible reasons for the blue-shift of the ESEEM frequencies could be the presence of a stray transverse magnetic field^18^ and the presence of non-secular Zeeman and hyperfine interactions^21^, which our theory does not consider (see Supplementary Note 1 for further details). Second, we found that the stretching exponent, determined from fits of the coherence decay is 2.3 in experiment, and 2.6 in theory. For the NV centre, our model yields 1.9, which is in a good agreement with previous analytical calculations^22^. Experimentally, in diamond, decay exponent ranging from 1.2 to 2.7 were reported^14^, depending on the sample and the *B*-field misalignment. Finally, the theoretical divacancy *T*_2_ times also saturate at a smaller *B* field than the experimental *T*_2_ times, for reasons we do not understand.

In this study, we considered the coherence of divacancy spin ensembles. However, the divacancy decoherence dynamics at the single-spin level is also of interest. In Supplementary Fig. 4, we show the variation of the divacancy single-spin *T*_2_ time in random nuclear spin environments compared with that of the NV centre in diamond. We find that the divacancy single-spin *T*_2_ ranges from 0.6 to 1.7 ms at a magnetic field of 11.5 mT, while it ranges from 0.4 to 1.4 ms at *B*=11.5 mT for the NV centre in diamond. Similar to the NV centre in diamond, the divacancy single-spin coherence dynamics could show a rich complex dynamics depending on individual local nuclear spin environments. Other important factors for the single-spin coherence in SiC may include the effects of strain, thermal, magnetic and electric inhomogeneities.

Our combined experimental and theoretical work lays a solid foundation to understand the robust divacancy spin coherence. The essential physics should apply to other potential spin qubits in SiC as well, thus providing a benchmark for future implementation of other spin qubits in this material^59^,^60^,^61^. Moreover, our model has implications beyond the crystal studied in this effort. The dynamics responsible for the coherence found in SiC, a binary crystal, may allow qubits in ternary and quaternary crystals to have even longer spin coherence times. For example, our results suggest that alloying the SiC lattice with larger elements such as Ge may further extend the coherence time of the divacancy spins. Since substitutional Ge would replace some ^29^Si atoms, it could serve as an alternative path to isotopic purification, especially for applications that require a large number of coherent spins. In addition, interesting host crystals with useful functionalities are normally found in binary or ternary crystals such as carbides, nitrides and oxides^59^,^62^. The piezoelectricity in AlN is one example. Complex oxides can exhibit exotic collective behaviours such as ferroelectricity, ferromagnetism and superconducting behaviour. Combining these collective degrees of freedom with coherent spin control in complex materials would be a promising route to hybrid quantum systems.

## Methods

### Experimental methods

As described in the main text, the 4*H*–SiC samples are high-purity semi-insulating wafers purchased from Cree, Inc (part number: W4TRD0R-0200). Since they contain ‘off-the-shelf' neutral divacancies, we dice them into chips and measure them without any further sample preparation. The SiC samples are 3–4 mm chips attached to coplanar microwave striplines with rubber cement. In turn, the microwave stripline is soldered to a copper cold finger, which is cooled by a Janis flow cryostat.

For ODMR measurements, we use a 300 mW, 1.27 eV (975 nm) diode laser, purchased from Thorlabs, Inc. 60 mW reaches the sample. We focus the laser excitation onto the sample using a 14 mm lens and collect the PL using that same lens. We then focus the collected PL onto an InGaAs photoreceiver, which was purchased from FEMTO, a German electronics manufacturer. Although we did ensemble measurement, it may be worth commenting on the count rates achieved in as-received samples. When single defects were considered in our previous study^32^, we observed count rates of 3–5 kcts. However, because we were using a lower efficiency measurement apparatus than the avalanche photodiodes used for diamonds, this should not be directly compared with the 20–30 kcts of a typical NV centre. To gate the laser during the Hahn-echo measurements, we use an acousto-optical modulator.

The radio frequency (RF) signals in this paper were generated by an Agilent E8257C source, whose output was gated using an RF switch (MiniCircuits ZASWA-2-50DR+). These signals were then combined, amplified to peak powers as high as 25 W (Amplifier Research 25S1G4A), and then sent to wiring in the cryostat. The RF and optical pulses were gated with pulse patterns generated by a digital delay generator (Stanford Research Systems DG645) and an arbitrary waveform generator (Tektronix AWG520). The phase of the Rohde & Schwartz signal was also controlled by the AWG520 through IQ modulation.

We used lock-in techniques to take all of the Hahn-echo data in this paper. Specifically, we alternated the phase of the final *π*/2 microwave pulse of the Hahn-echo sequence between +π/2 and −π/2. This alternation causes the spin coherence, at the end of the Hahn-echo sequence, to be projected alternatively to opposite poles of the *m*_s_=+1/*m*_s_=+0 Bloch sphere. Because the (*kk*)-divacancy's PL from the *m*_s_=+1 pole of the Bloch sphere is stronger than that from the *m*_s_=+0 pole, this alternation induces a change in PL (ΔPL) between the two pulse sequences. Without spectrally filtering the PL, the ODMR contrast (ΔPL/PL) is roughly 0.5%. When spectrally filtering the PL (which we did not do in this work), the ODMR contrast is 20% for the (*kk*)-divacancy. To transform the ΔPL signals to a spin coherence measurement, we simply normalized the ΔPL−*t*_free_ traces, by dividing them by the maximum of the ΔPL trace.

### Theoretical methods

To calculate the Hahn-echo coherence of the (*kk*)-divacancy in 4*H*–SiC and the NV centre in diamond, we considered a central spin model in which an electron spin with total spin 1 is coupled to an interacting nuclear spin bath through the secular electron-nuclear hyperfine interaction. Given the dilute nature of the nuclear spin density both in 4*H*–SiC (4.7% of ^29^Si and 1.1% of ^13^C) and diamond (1.1% of ^13^C), we only considered the direct dipole–dipole interaction for the nuclear–nuclear spin coupling. We calculated the full time-evolution of the combined qubit and nuclear-bath system, and computed the off-diagonal elements of the reduced qubit density matrix by tracing out the bath degrees of freedom at the end of the Hahn-echo sequence (*π*/2 pulse−*t*_free_/2−*π* pulse−*t*_free_/2−echo). We considered randomly generated nuclear spin bath ensembles. A heterogeneous nuclear spin bath in 4*H*–SiC has ∼1,500 nuclear spins within 5 nm from the divacancy site, while the nuclear spin bath of diamond has ∼1,000 nuclear spins within 5 nm form the NV centre. We used the cluster-correlation expansion theory to systematically approximate the coherence function. Further details are found in Supplementary Notes 1–3.

### Code availability

The codes that were used in this study are available upon request to the corresponding author.

### Data availability

The data that support the findings of this study are available upon request to the corresponding author.

## Additional information

**How to cite this article:** Seo, H. *et al*. Quantum decoherence dynamics of divacancy spins in silicon carbide. *Nat. Commun.* **7,** 12935 doi: 10.1038/ncomms12935 (2016).

## Supplementary Material

Supplementary Information Supplementary Figures 1-8, Supplementary Table 1, Supplementary Note 1-3 and Supplementary References
