# Supplementary material for: Quantum decoherence dynamics of divacancy spins in silicon carbide
Source: Nat Commun. 2016 Sep 29;7:12935. doi: 10.1038/ncomms12935 (PMC5056425; doi:10.1038/ncomms12935)
Supplement: Supplementary Information — Supplementary Figures 1-8, Supplementary Table 1, Supplementary Note 1-3 and Supplementary References [file ncomms12935-s1.pdf]

Supplementary Figures

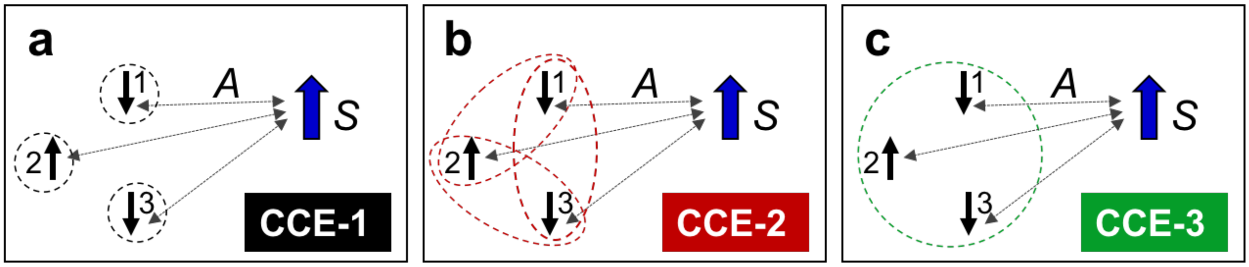

Supplementary Figure 1. CCE method. A system of electron spin (*S*) interacting with three nuclear spins (1,2,3) is considered. In CCE-1 (a), each nuclear spin is treated independently and it only interacts with the electron spin (*S*) through the hyperfine coupling (*A*). In CCE-2 (b) and CCE-3 (c), irreducible pair and triple correlations (see text) from possible nuclear spin pairs and triples, respectively, are recursively added to the single-correlation terms calculated in CCE-1. As there are only three nuclear spins in the bath, CCE-3 provides an exact solution of the electron spin coherence.

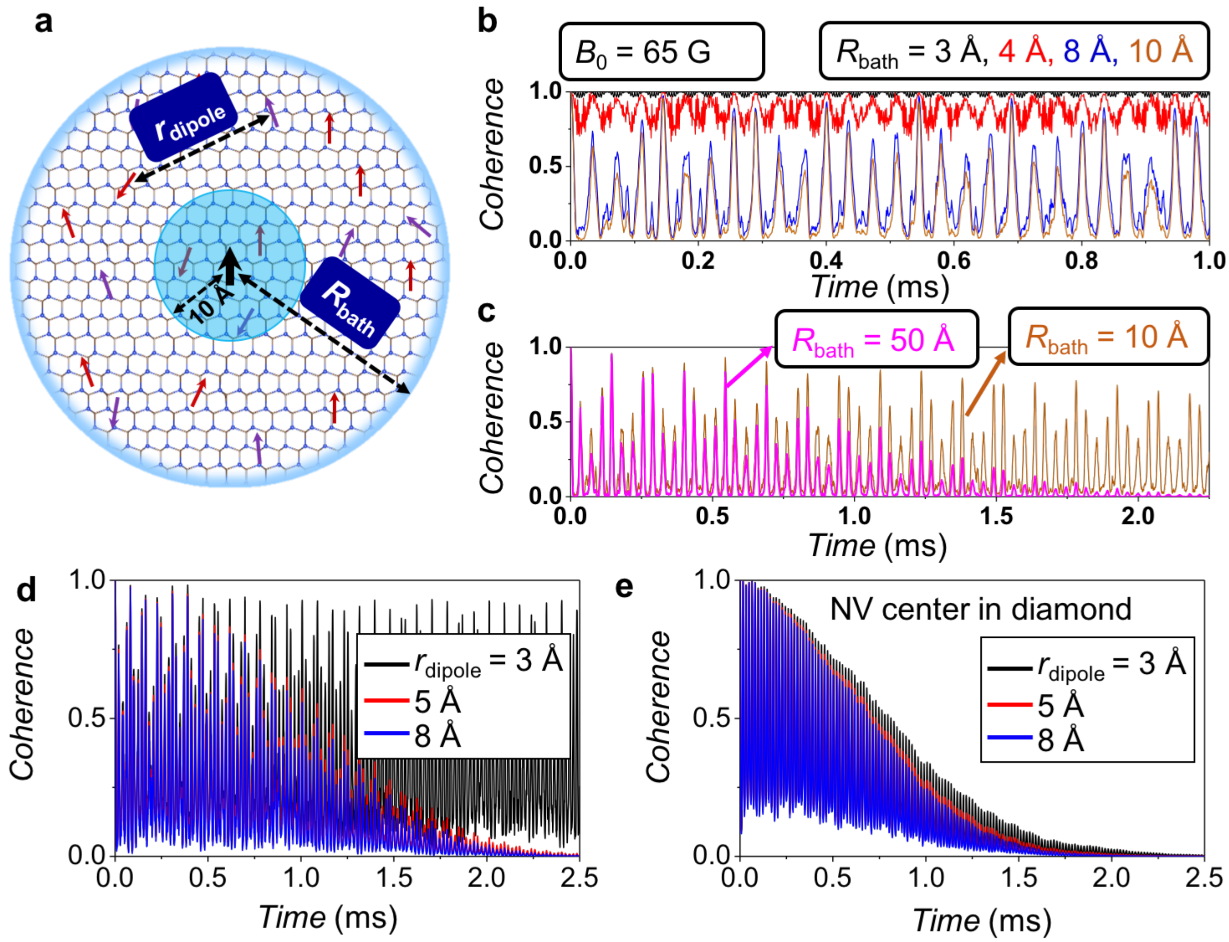

Supplementary Figure 2. Numerical convergence tests of the Hahn-echo coherence. (a) Schematic of a divacancy spin qubit (black arrow in the middle) coupled to a heterogeneous nuclear spin bath in 4*H*-SiC. Red arrows represent ^{29}Si nuclear spins (4.7%, I_{Si} = ½), while ^{13}C nuclear spins (1.1%, I_C = ½) are denoted by purple arrows. Two numerical parameters, R_{bath} and r_{dipole} are a cutoff radius for defining the bath size and a cutoff distance for the dipolar coupling between two nuclear spins, respectively. (b) The divacancy coherence at a magnetic field of 65 G at the CCE-2 level of theory calculated for four different bath sizes: black for R_{bath} = 3 Å, red for 4 Å, blue for 8 Å, and brown for 10 Å. The coherence oscillation is mainly determined by nuclear spins within R_{bath} = 10 Å, defining a strong coupling regime schematically shown as a blue area in (a). (c) The Hahn-echo coherence as in (b), but for R_{bath} = 50 Å and 10 Å, showing that nuclear spins beyond R_{bath} = 10 Å are mainly responsible for the coherence decay. The coherence function is numerically converged with R_{bath} = 50 Å. (d), (e), The coherence of the divacancy (d) and the NV center (e) at a magnetic field of 115 G calculated for three different r_{dipole}.

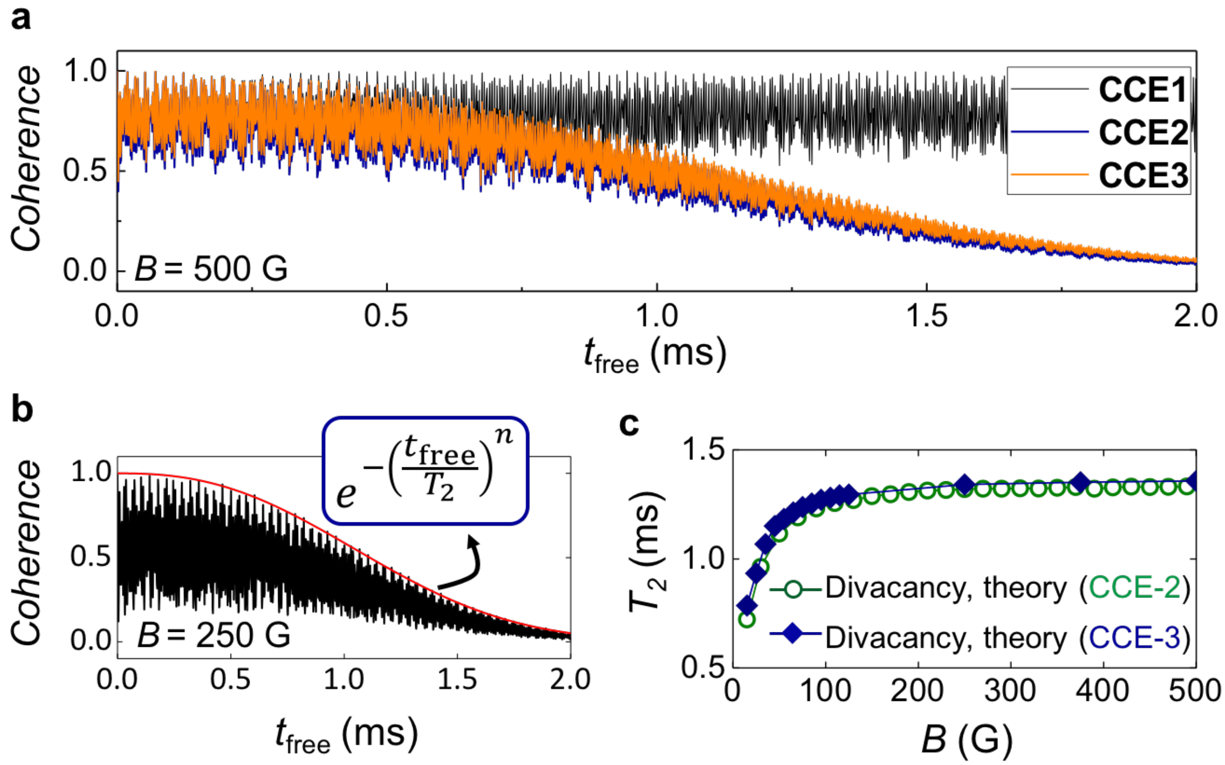

Supplementary Figure 3. Numerical convergence of the coherence with respect to the CCE order. (a) The coherence of the divancay at a magnetic field of 500 G calculated at the CCE-1 (black), CCE-2 (blue), and CCE-3 (orange) levels of theory. CCE-3 does not give significant correction to the CCE-2 results, implying that the CCE-2 approximation provides numerically converged results. (b) Fitting of the divacancy coherence at B = 250 G with a stretched exponential function having two parameters: the Hahn-echo coherence time T_2 and an stretching exponent n. (c) T_2 of the divacancy as a function of static magnetic field at the CCE-2 and CCE-3 levels of theory. The CCE-2 and CCE-3 results are in excellent agreement with each other, further providing the numerical validity of CCE-2.

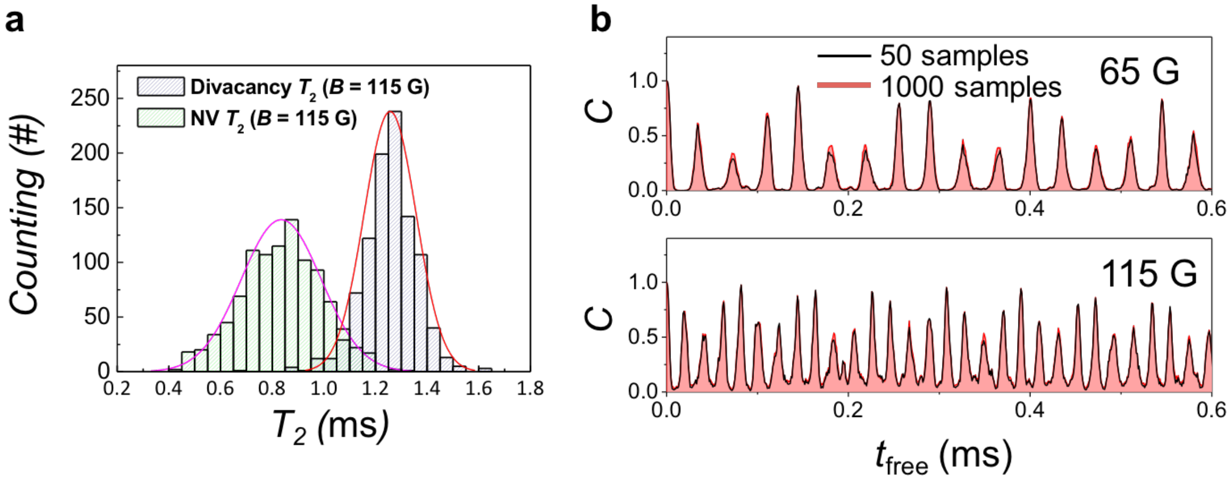

Supplementary Figure 4. Ensemble statistics. (a) Distribution of T_2 of the divacancy ensemble in 4*H*-SiC and the NV ensemble in diamond at a static magnetic field of 115 G. Red curves are normal distribution fit of the histograms. (b) Direct comparison of the coherence of the divacancy in 4*H*-SiC averaged over 50 different random nuclear spin baths (Black curve) to the coherence averaged over 1000 nuclear spin baths (Filled red curve) at B = 65 G (up) and B = 115 G (down).

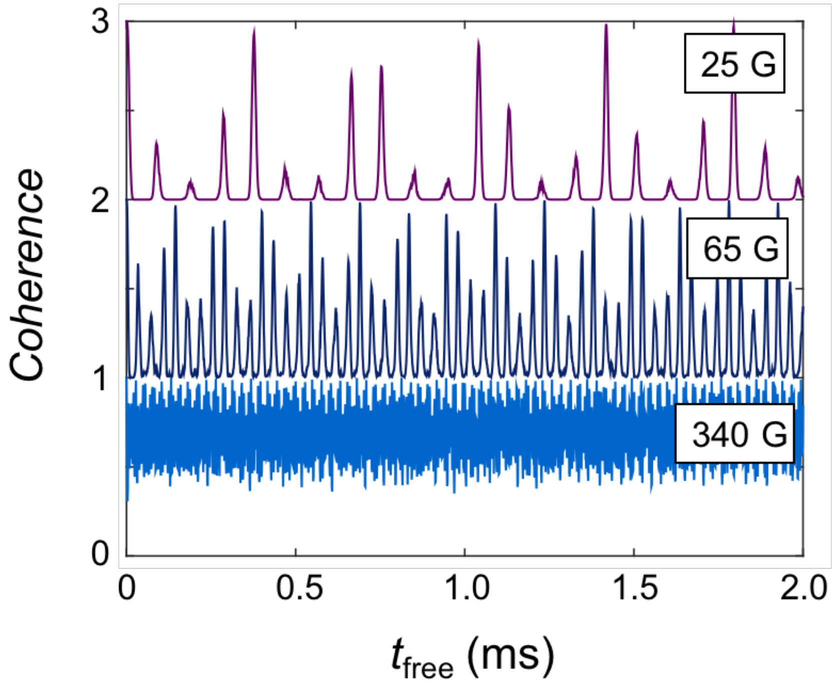

Supplementary Figure 5. ESEEM spectra calculated within CCE-1. Analytical expression in Supplementary Equation 16 is used along with the same numerical strategy used for the results in Figure 2 (b) in the main article. The CCE-1 calculations reproduce all the features in the coherence functions in Figure 2 (b) of the main article except for the overall envelop decay.

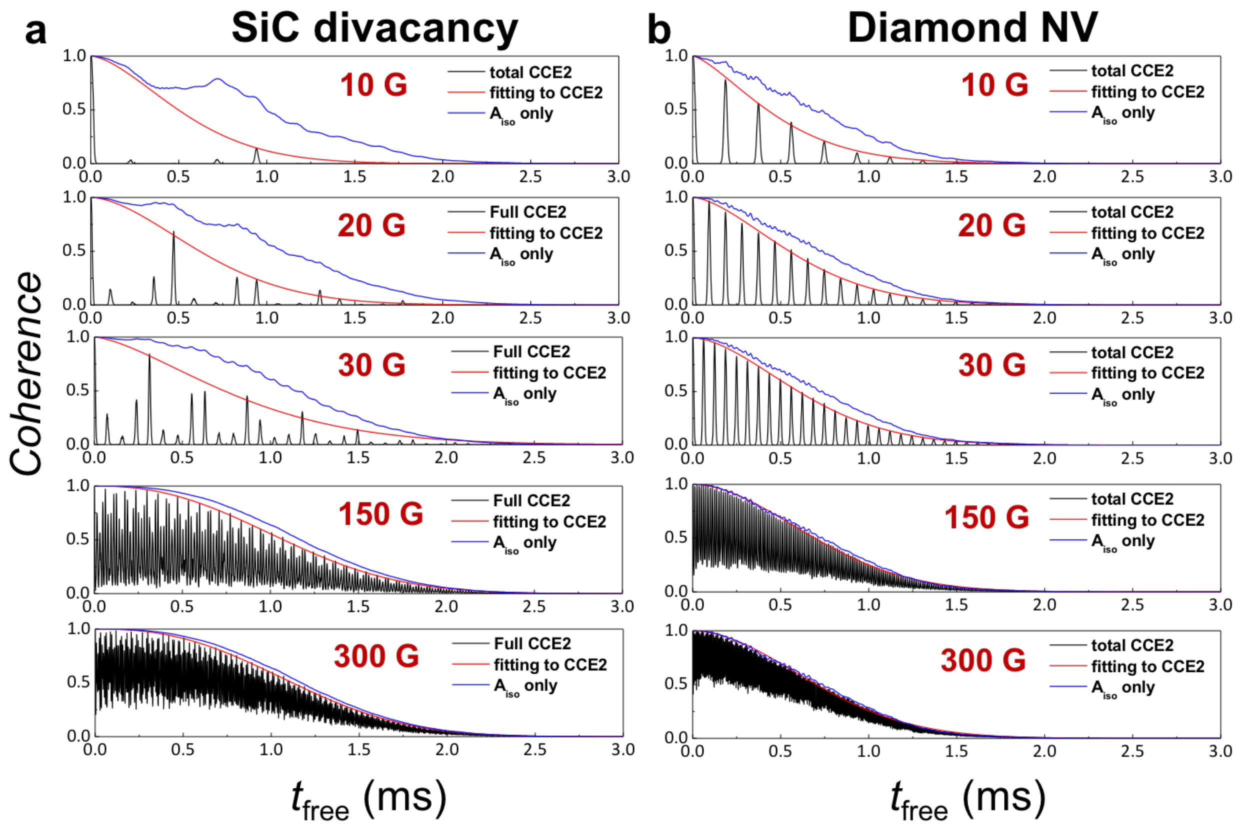

Supplementary Figure 6. Pseudo-secular hyperfine field induced coherence decay. The Hahn-echo coherence of the divacancy in 4*H*-SiC (a) and the NV center in diamond (b) at several magnetic fields calculated with the full hyperfine coupling (A_{i}, B_{ix}, and B_{iy} in Supplementary Equation 7) (black curve) and without anisotropic hyperfine coupling (blue curve, A_{i} only). The red curve is a fit to the full CCE-2 curve and the difference between the red curve and the A_{i}-only blue curve is the contribution from the pseudo-secular hyperfine interactions to the coherence decay, which becomes negligible at a large magnetic field beyond 100 G for both divacancy and NV.

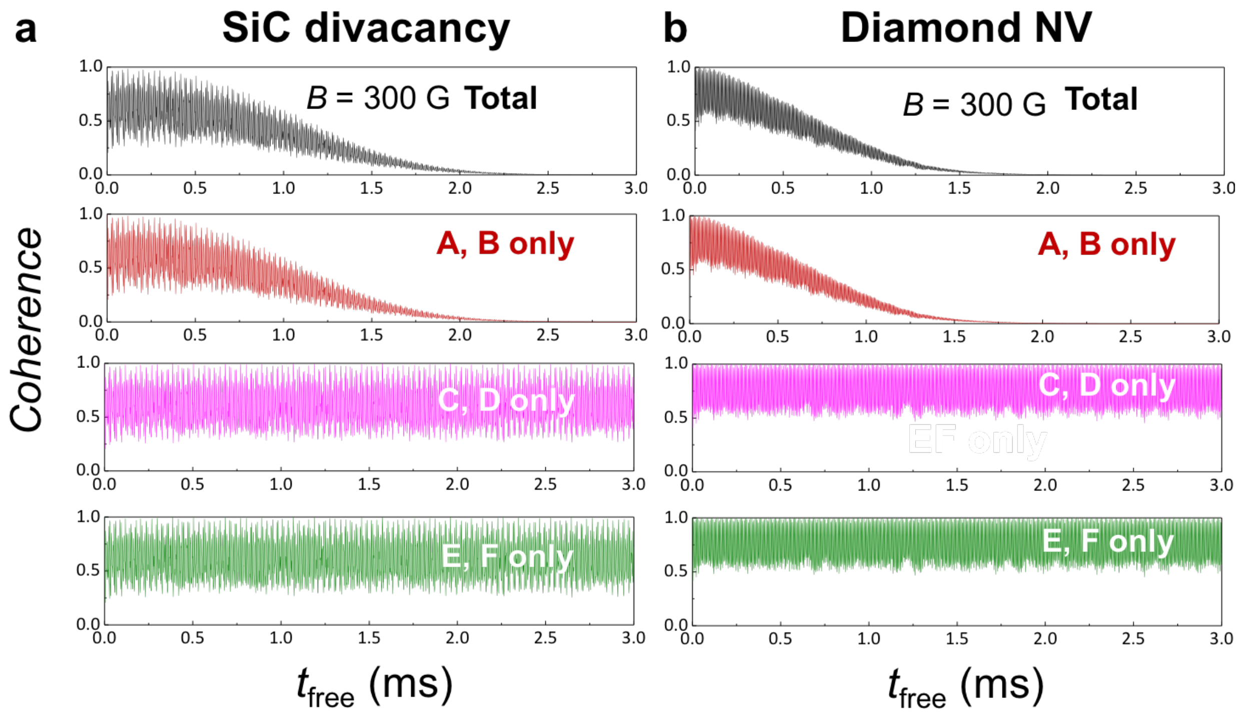

Supplementary Figure 7. Central spin decoherence induced by nuclear spin flip-flop transitions. The full Hahn-echo coherence of the divacancy in 4*H*-SiC (a) and the NV center in diamond (b) at B = 300 G at the top (black curve) compared to those calculated only with the A and B term (red curve), the C and D terms (cyan curve) and the E and F terms (green curve) of the nuclear dipole-dipole coupling Hamiltonian shown in Supplementary Equation 18. At a large magnetic field above B = 100 G, the nuclear flip-flop transitions induced by the A and B terms are the main cause of the coherence decay for both divacancy and NV qubits, while the transitions induced by the other terms are fully suppressed.

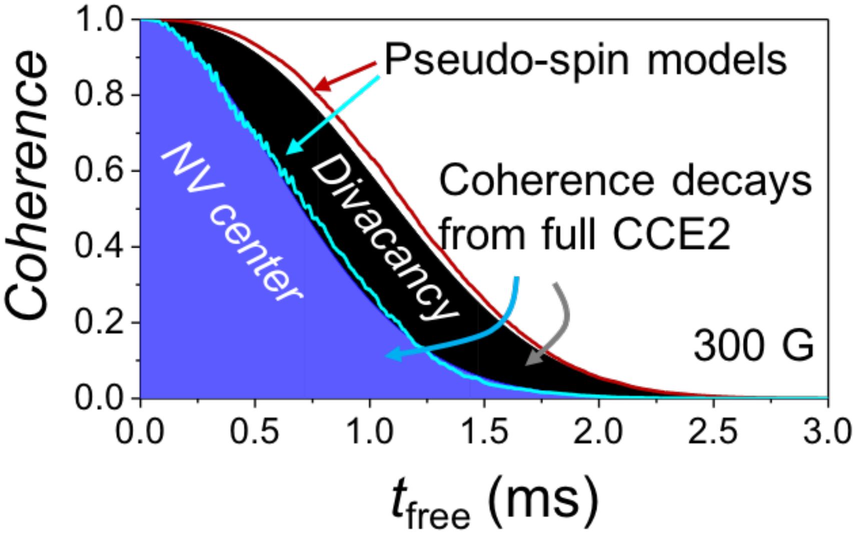

Supplementary Figure 8. Pseudo-spin model of decoherence. The coherence decay of the divacancy in 4*H*-SiC (filled black curve) and the NV center in diamond (filled blue curve) from the full CCE calculations, for which only the envelop decay is shown for clarity. The red and the cyan curves are the coherence decay curves of the divacancy and the NV center, respectively, calculated by using the pseudo-spin model shown in Supplementary Equation 23.

Supplementary Table

Defect spin qubit	B_0 (G)	Number of samples	T_2 average (ms)	T_2 STDEV (N-1) (ms)	n average (ms)	n STDEV (N-1) (ms)
(kk)-divacancy in 4H-SiC	65	50	1.17	0.18	2.26	0.37
		100	1.19	0.15	2.30	0.32
		1000	1.18	0.14	2.27	0.32
	115	50	1.26	0.11	2.42	0.33
		100	1.27	0.10	2.47	0.26
		1000	1.26	0.10	2.45	0.25
NV center in diamond	65	50	0.816	0.182	2.178	0.492
		100	0.807	0.168	2.222	0.487
		1000	0.796	0.166	2.220	0.464

Supplementary Table 1. Computed ensemble-averaged T_2 and n of the divacancy qubit in 4H-SiC and the NV center in diamond along with their standard deviation (STDEV) computed with the (N-1) method, where N is the number of samples in ensemble.

Supplementary Note 1. Theoretical calculations of decoherence dynamics

Quantum bath approach to qubit decoherence.

To calculate the decoherence dynamics of divacancy spin qubits in 4H-SiC, we use a microscopic quantum bath approach, in which a combined qubit and bath system is considered as a closed quantum system[1]. The phase information of a qubit at an arbitrary time t is encoded in the off-diagonal element of the reduced density matrix, for which the bath degrees of freedom are traced out. Suppose that a combined qubit and bath system is initialized at t=0 as a product state as follows:

$$|\Psi(0)\rangle = \frac{1}{\sqrt{2}}(|1\rangle + |0\rangle)\otimes|\mathcal{B}(0)\rangle, \tag{1}$$

where $|1\rangle$ and $|0\rangle$ are up and down states of the qubit, respectively, and $|\mathcal{B}(0)\rangle$ is an initial state of the bath. In the course of time evolution, the bath state may be entangled with the qubit state:

$$|\Psi(\tau)\rangle = \frac{1}{\sqrt{2}}\left(|0\rangle\otimes|\mathcal{B}^{(0)}(\tau)\rangle + |1\rangle\otimes|\mathcal{B}^{(1)}(\tau)\rangle\right). \tag{2}$$

The off-diagonal element of the reduced density matrix is then given as an overlap between the two bath states ($|\mathcal{B}^{(0)}(\tau)\rangle$ and $|\mathcal{B}^{(1)}(\tau)\rangle$). Therefore, in order to use the quantum-bath method, we need to identify the dominant bath degrees of freedom of a given system and calculate the bath evolution conditioned on qubit states.

It has been established for the nitrogen-vacancy (NV) center in diamond that the main source of the spin decoherence is its coupling to the ^{13}C nuclear spin bath (1.1% abundance, I_C = 1/2) and other paramagnetic defect centers such as N impurities (P1 centers) in the lattice[2]. The later can be controlled by a chemical purification process and the longest Hahn-echo ensemble coherence time (T_2) of the NV centers in high-purity diamond has been measured to be 0.63 ms[3]. A similar argument can be applied to the divacancy qubits in 4H-SiC except that the nuclear spin bath of 4H-SiC is a heterogeneous one having both naturally occurring ^{29}Si isotopes (4.7%, I_{Si}=1/2) and ^{13}C isotopes. Other paramagnetic defects might be generated during sample preparation. We note, however, that a defect density in our samples is very low as described in the main text. The divacancy density is approximately 10^{12} cm^{-3} [4] and an unintentional dopant density is also very low (5×10^{13} cm^{-3})[5]. Considering a paramagnetic defect density of 10^{13} ~ 10^{14} cm^{-3}, there may be one or two paramagnetic impurities within 1000 ~ 3000 Å from a divacancy qubit in 4H-SiC with dipolar coupling strengths ranging from 50 Hz to 2 Hz, while there are already ~ 10 nuclear spins even within 10 Å with electron-nuclear dipolar coupling strengths ranging from 0.1 MHz to 0.01 MHz. Thus, contribution from paramagnetic defect centers to the divacancy decoherence may be negligible in our SiC

samples and we only focus on the effect of the fluctuating nuclear spin bath due to the nuclear-nuclear dipolar interactions.

It is worth discussing about possible temperature effects on nuclear and electron spins in SiC and diamond. In principles, electron and nuclear spins can be randomly flipped at a finite temperature, inducing qubit decohernece[6]. Temperature-induced flipping of nuclear and electron spins can be characterized by nuclear and electronic spin-lattice relaxation times, $T_{1,\mathrm{n}}$ and $T_{1,\mathrm{e}}$, respectively. It has been found that the $T_{1,\mathrm{n}}$ times in SiC and diamond are extremely long exceeding several hours owing to the lack of efficient nuclear spin-lattice coupling mechanism[7,8]. The time scale of the NV center coherence and that of the divacancy qubits has been measured to be ~ millisecond, meaning the $T_{1,\mathrm{n}}$-induced nuclear spin flipping to be negligible in this time scale. $T_{1,\mathrm{e}}$-induced relaxation of a central electron qubit maybe another issue in SiC and diamond at a finite temperature as the qubit's T_2 time is ultimately limited by $2T_{1,\mathrm{e}}$[2,9]. Temperature-dependent $T_{1,\mathrm{e}}$ times of the NV center and the divacancy have been measured to be ranging from 6×10^{-3} s (at T = 300 K) to 2×10^{2} s (at T = 10 K)[10] and from 6.2×10^{-4} s (T = 250 K) to 2.0×10^{-2} s (T = 20 K)[4], respectively. Therefore, we also ignore the $T_{1,\mathrm{e}}$-induced relaxation effect on the central qubit decoherence in SiC and diamond at T = 20 K.

Spin Hamiltonian and Hahn-echo coherence function.

Considering the fluctuating nuclear spin bath as a main source of the divacancy decoherence, we can write down the spin Hamiltonian as $\mathcal{H}_{\mathrm{total}} = \mathcal{H}_{\mathrm{S}} + \mathcal{H}_{\mathrm{B}} + \mathcal{H}_{\mathrm{S-B}}$, where $\mathcal{H}_{\mathrm{S}}$ and $\mathcal{H}_{\mathrm{B}}$ are terms for the qubit and the bath under a static magnetic field ($\vec{B} = B_0\hat{z}$), respectively, while $\mathcal{H}_{\mathrm{S-B}}$ accounts for the hyperfine coupling between the qubit and the bath[9]. Each term can be written as follows:

$$\mathcal{H}_{\mathrm{S}} = -\gamma_e \hbar \vec{B} \cdot \vec{S} + \Delta S_z^2, \tag{3}$$

$$\mathcal{H}_{\mathrm{B}} = -\vec{B} \cdot \sum_i \gamma_i \hbar \vec{I}_i + \mathcal{H}_{n-n}, \tag{4}$$

$$\mathcal{H}_{\mathrm{int}} = \vec{S} \cdot \sum_i \overleftrightarrow{A}_i \cdot \vec{I}_i, \tag{5}$$

where γ_e and γ_i (i = C or Si) are the gyromagnetic ratios of electron and nuclear spins of ^{29}Si and ^{13}C isotopes, respectively, and they are given as γ_{e}= -1.761×10^{11} rad s^{-1} T^{-1}, γ_{Si}= -5.319×10^{7} rad s^{-1} T^{-1} and γ_{C}= 6.728×10^{7} rad s^{-1} T^{-1}. The second term in $\mathcal{H}_S$ is the zero-field splitting tensor splitting the m_s=0 and m_s=±1 sublevels of the electron spin (S = 1) and it has been measured to be 1.305 GHz for the *(kk)*-divacancy spin in 4*H*-SiC[11]. $\mathcal{H}_{\mathrm{n-n}}$ is the magnetic dipole-dipole coupling between two nuclear spins and it is given by:

$$\mathcal{H}_{\text{n-n}} = \frac{\mu_0}{4\pi}\sum_{i<j}\gamma_i\gamma_j\hbar^2\left(\frac{\vec{I}_i\cdot\vec{I}_j}{r_{ij}^3} - \frac{3(\vec{I}_i\cdot\vec{r}_{ij})(\vec{I}_j\cdot\vec{r}_{ij})}{r_{ij}^5}\right), \tag{6}$$

where r_{ij} is the distance between the nuclear spin I_i and I_j. The hyperfine tensor ($\overleftrightarrow{A_i}$) that couples the electron spin to the i-th nuclear spin in the bath may have two parts: the isotropic Fermi contact interaction and the anisotropic dipole-dipole interaction[9]. The Fermi contact term is mainly derived from the overlap between the defect's electron spin density and the nuclear spin under consideration. We note, however, that the defect spin density is highly localized in space owing to the localized nature of the carbon sp^3 dangling bonds[11]. Thus, the Fermi contact term may become negligible compared to other energy scales in the Hamiltonian beyond three to four nearest neighboring sites. In this study, we ignore the Fermi contact term. In addition, we ignore the off-diagonal non-secular S_x and S_y terms in the anisotropic dipolar coupling because the zero-field splitting of GHz order of magnitude and the large difference between the electron and nuclear gyromagnetic ratios would make the hyperfine-induced flipping of the electron spin almost impossible in the time-scale that we are interested in. This 'secular approximation' is also a valid approximation if the spin-lattice relaxation time $T_{1,e}$ is much larger than the pure-dephasing time T_2[9], which is our case[4,10]. The final form of the hyperfine interaction is written as follows:

$$\mathcal{H}_{\text{int}} = S_z\sum_i \vec{A}_i\cdot\vec{I}_i = \sum_i\left(B_{ix}I_{ix}S_z + B_{iy}I_{iy}S_z + A_iI_{iz}S_z\right), \tag{7}$$

where $\vec{A}_i$ is the hyperfine field for the ith nuclear spin I_i, consisting of secular A_i hyperfine coupling and pseudo-secular B_{ix} and B_{iy} hyperfine couplings. We note that the hyperfine field is only active when the electron spin is not in the $m_s = 0$ state. We also observe that the secular coupling term gives rise to the Zeeman frequency shift for a nuclear spin while the pseudo-secular coupling terms can flip the nuclear spin, thus creating a fluctuation in the nuclear spin bath at low magnetic fields. Within the secular approximation, the total Hamiltonian commutes with the S_z operator and the electron spin is preserved, allowing us to project the total Hamiltonian on the electron spin basis. As a result, we obtain the following pure-dephasing Hamiltonian[1]:

$$\mathcal{H}_{\text{total}} = \sum_{m_s=-1}^{+1} |m_s\rangle\langle m_s| \otimes \mathcal{H}_{m_s}, \tag{8}$$

where $\mathcal{H}_{m_s}$ is the bath Hamiltonian conditioned on the electron spin sub-level m_s.

$$\mathcal{H}_{m_s} = \omega_{m_s} + \mathcal{H}_B + m_s\sum_i \vec{A}_i\cdot\vec{I}_i, \tag{9}$$

where ω_{m_s} is the energy spectrum of the electron spin. We note that the same Hamiltonian and the same approximation are applied to the NV center in diamond except that the C lattice only has ^{13}C nuclear spins and there is ^{14}N-derived nuclear spin (I_N=1) associated with the NV center.

The coherence function, the off-diagonal element of the reduced density matrix, can be formally written as:

$$\mathcal{L}(t) \equiv \frac{tr[\rho_{\text{tot}}(t)S_+]}{tr[\rho_{\text{tot}}(0)S_+]}, \tag{10}$$

where $S_+ = S_x + iS_y$ is the electron spin raising operator and ρ_{tot} is the density of operator of the combined qubit (ρ_S) and bath (ρ_B) system. At $t = 0$, we assume that the system is initialized as the product state as $\rho_{\text{tot}}(0) = \rho_s(0) \otimes \rho_B(0)$ and it evolves in time as $\rho_{\text{tot}}(t) = \mathcal{U}(t)\rho_{\text{tot}}(0)\mathcal{U}^\dagger(t)$, where $\mathcal{U}(t)$ is the Hahn-echo propagator[9]. We employ the assumption of piecewise constant Hamiltonian, in which the Hahn-echo propagator in the rotating frame breaks into a $\pi/2$-pulse bringing the initial down-state ($m_s = 0$) into a superposition of the up ($m_s = +1$) and down states, followed by a free-evolution under a given static magnetic field for $t_{\text{free}}/2$, an ideal π-pulse ($P_\pi = -i\sigma_x$), and another $t_{\text{free}}/2$ free-evolution under static B-field, subsequently. Noting that the free evolution operator is block-diagonal as the pure-dephasing Hamiltonian in Supplementary Equation 8 does not mix the up and down states of the electron spin, one can finally write down the Hahn-echo coherence as:

$$\mathcal{L}(t_{\text{free}}) = tr[\rho_{\text{tot}}(t_{\text{free}})S_+] = tr_B\left[\mathcal{U}_-^\dagger\mathcal{U}_+^\dagger\mathcal{U}_-\mathcal{U}_+\rho_{\text{Bath}}(0)\right] = \sum_{\mathcal{J}} \mathcal{P}_{\mathcal{J}} \langle\mathcal{J}|\mathcal{U}_-^\dagger\mathcal{U}_+^\dagger\mathcal{U}_-\mathcal{U}_+|\mathcal{J}\rangle, \tag{11}$$

where $\rho_{\text{Bath}}(0) = \sum \mathcal{P}_{\mathcal{J}}|\mathcal{J}\rangle\langle\mathcal{J}|$. $\mathcal{U}_+ = e^{-(i/\hbar)(\mathcal{H}_B + \sum_i \vec{A}_i \cdot \vec{I}_i)t_{\text{free}}/2}$ and $\mathcal{U}_- = e^{-(i/\hbar)\mathcal{H}_B t_{\text{free}}/2}$ are free bath propagators conditioned on the up and down states of the electron spin, respectively. We note that at T = 20 K, the nuclear spin bath is almost completely thermalized, making the initial nuclear spin bath density matrix to be the identity.

Supplementary Note 2. Cluster correlation expansion

Concepts and numerical implementation.

Supplementary Equation 11 formally allows for calculating the coherence of the divacancy and the NV qubits. However, the direct matrix calculations are still an unfeasible task as a large number of nuclear spins are involved. For instance, there are around 1500 nuclear spins in 4*H*-SiC and 1000 nuclear spins in diamond within 5 nm from a divacancy qubit and a NV center, respectively, leading to a matrix dimension of 2^{1000} to 2^{1500} to be solved. Recently developed cluster correlation expansion (CCE) technique[12,13] enables a systematic approximation to the coherence function. The basic concept of CCE is schematically shown in Supplementary Figure 1. Suppose a spin qubit is coupled to a bath of three nuclear spins. The simplest approximation is to ignore all the interactions between the nuclear spins and treat them independently, yielding a CCE-1 coherence function that is a product of all the 'single-correlation' terms as schematically shown in Supplementary Figure 1 (a).

$$\mathcal{L}_1(t_{\text{free}}) = \prod_i \tilde{\mathcal{L}}_i(t_{\text{free}}) = \prod_i \mathcal{L}_i(\tau)/\tilde{\mathcal{L}}_0, \tag{12}$$

where i is an index for nuclear spins (i=1,2,3) and $\tilde{\mathcal{L}}_0$ is a normalization constant or 'empty-correlation' term. Apparently, the independent nuclear spin model cannot capture dipole-dipole induced bath fluctuations[14]. The next-order approximation would be to include two-body or pair-correlation effects (see Supplementary Figure 1 (b)):

$$\mathcal{L}_2(t_{\text{free}}) = \prod_i \tilde{\mathcal{L}}_i(t_{\text{free}}) \prod_{\{i,j\}} \tilde{\mathcal{L}}_{i,j}, \tag{13}$$

where $\tilde{\mathcal{L}}_{i,j} = \mathcal{L}_{i,j}(t_{\text{free}})/(\tilde{\mathcal{L}}_i\tilde{\mathcal{L}}_j)$. Note that if two nuclear spin pairs share one nuclear spin in common (see Supplementary Figure 1), the dipole-dipole induced transitions of the two pairs may be correlated to each other. This three-body correlation can be captured at the next CCE-3 level of theory:

$$\mathcal{L}_3(t_{\text{free}}) = \prod_i \tilde{\mathcal{L}}_i(t_{\text{free}}) \prod_{\{i,j\}} \tilde{\mathcal{L}}_{i,j} \prod_{\{i,j,k\}} \tilde{\mathcal{L}}_{i,j,k} \tag{14}$$

where $\tilde{\mathcal{L}}_{i,j,k} = \mathcal{L}_{i,j,k}(t_{free})/(\tilde{\mathcal{L}}_i\tilde{\mathcal{L}}_j\tilde{\mathcal{L}}_k)/(\tilde{\mathcal{L}}_{i,j}\tilde{\mathcal{L}}_{j,k}\tilde{\mathcal{L}}_{i,k})$. In this simple example of the 3-nuclear-spin model, we remark that the CCE-3 coherence function in Supplementary Equation 14 is the same as the exact coherence function, i.e. $\mathcal{L}_3(t_{\text{free}}) = \mathcal{L}_{1,2,3}(t_{\text{free}})$. This means that for any possible nuclear spin baths, CCE expansion provides the exact solution when the expansion includes the largest possible nuclear spin clusters (i.e. the entire nuclear spin bath). For practical calculations, the expansion would stop at a certain cluster size *N*, and the CCE-*N* expansion is given by:

$$\mathcal{L}_N(\tau) = \prod_{C \subseteq \{1,2,3,\ldots,N\}} \tilde{\mathcal{L}}_C(\tau), \tag{15}$$

where all the irreducible cluster correlations up to clusters with *N* nuclear spins being included. *N* for a specific system can be determined by calculating the numerical convergence with respect to *N*, which will be further discussed later in this article.

We used C/C++ and the Eigen3 library[15] to implement the CCE method. We created orthorhombic supercells of 4*H*-SiC and C diamond and placed a (*kk*)-divacancy defect and a NV center in the middle of the SiC and C supercells, respectively. We used experimentally determined lattice structures of 4*H*-SiC and diamond and the *c*-direction of the supercells are aligned with the C_{3v}-axis of the defects: (0001) for the (*kk*)-divacancy and (111) for NV, along which static magnetic field is applied. The presence of nuclear spins in the lattices naturally occurring from ^{13}C and ^{29}Si isotopes were simulated by randomly placing ^{13}C and ^{29}Si nuclear spins at 1.1% and 4.7% concentrations in the supercells. The same strategy was used to generate multiple supercells for creating an ensemble of random heterogeneous nuclear spin baths of ^{29}Si and ^{13}C in 4*H*-SiC and an ensemble of homogeneous nuclear spin baths with ^{13}C for C diamond. The size of the supercell and the number of supercells in an ensemble have been systematically determined by checking the numerical convergence with respect to the bath size and the ensemble average, which will be described in the next section.

Numerical convergence.

There are a number of numerical parameters that need to converge in our CCE calculations: (1) size of the nuclear spin bath (R_{bath}), (2) the largest dipole-dipole interaction distance between two nuclear spins (r_{dipole}), and (3) the CCE expansion order. In this section, we discuss each of them and their physical implications. All calculations done in this section are ensemble-averaged over 50 nuclear spin bath samples. Convergence of the ensemble average will be discussed in the next section. In addition, we only discuss results for the divacancy in 4*H*-SiC for simplicity. The convergence test results for NV in diamond will be discussed briefly at the end of this section.

In the supercell geometry discussed in 2-a, a central $S = 1$ spin qubit (either divacancy or NV) is coupled to a random nuclear spin bath mainly through the electron-nuclear dipolar coupling, which decays as $1/R^3$ where R is the distance between the electron spin and a nuclear spin under consideration. Thus, beyond a certain cutoff radius defined as R_{bath} the *e*-*n* coupling may become negligible, defining the bath size as shown in Supplementary Figure 2 (a). In Supplementary Figure 2 (b), we calculate the divacancy coherence function for different bath sizes under a static magnetic field of 65 G at the CCE-2 level of theory. As noted in the main text the divacancy coherence function comprises of the electron spin echo envelop

modulation (ESEEM) and the overall decay. In Supplementary Figure 2 (b), the ESSEM pattern rapidly emerges as the bath size increases from 3 Å (only including the nearest neighboring sites) to 8 Å. A further increase of the bath size to 10 Å does not significantly change the oscillation pattern, indicating that the origin of the ESSEM spectrum is the strong hyperfine coupling with ~ 10 nuclear spins within 10 Å. In addition, we find that nuclear spins beyond the strong coupling regime (See Supplementary Figure 2 (a)) is mainly responsible for the coherence decay as shown in Supplementary Figure 2 (c), which compares the divacancy coherence function calculated with the small bath of R_{bath} = 10 Å to that of a larger nuclear spin bath of R_{bath} = 50 Å. In addition, we note that the coherence function does not change as we vary the bath size from 40 Å to 60 Å. Therefore, we set R_{bath} = 50 Å to be our cutoff radius for the nuclear spin bath that the central (*kk*)-divacancy is coupled with. This observation also lays down a solid ground for our quantum-bath approach to decoherence, which assumes that the combined qubit and bath system form a closed quantum system. Our numerical convergence tests show that this assumption is self-consistently valid for the (*kk*)-divacancy coupled with nuclear spins within R_{bath} = 50 Å.

In principles, CCE calculations at a given expansion order, e.g. CCE-2, should involve all possible pairs of nuclear spins. However, some remote nuclear spins would not interact strong enough to contribute to the coherence decay because the nuclear dipole-dipole coupling scales as $1/r^3$, where r is the distance between two nuclear spins. Thus, we introduced a cutoff distance, r_{dipole} and we treat two nuclear spins as independent spins if they are separated by more than r_{dipole}. We perform CCE-2 calculations for various r_{dipole} values and we found that the numerical convergence is achieved for r_{dipole} = 6 Å and we used r_{dipole} = 8 Å for all calculations for this work.

Practical CCE calculations are terminated at a certain CCE order known as the CCE-*N* approximation, where *N* indicates the number of nuclear spins in the largest cluster considered. The order of CCE calculations should depend on the problem under investigation and should be determined by checking the numerical convergence with respect to the CCE order. In Supplementary Figure 3 (a), we show representative coherence functions of the divacancy qubit calculated at difference CCE orders. We found that the CCE-2 and CCE-3 coherence functions show negligible differences, indicating that CCE-2 calculations provide full numerical convergence. We further verify the numerical convergence by comparing T_2 as a function of static magnetic field in Supplementary Figure 3 (c). We note that the CCE-2 and CCE-3 results of T_2 show negligible difference across a wide range of magnetic field.

The validity of the CCE-2 approximation on our problem could be understood by considering that our nuclear spin concentration in the lattice is very low and the nuclear dipole-dipole interaction decays fast as $1/r^3$. Given our r_{dipole} of around 6 to 8 Å, it is hard to form a significant number of strongly coupled

nuclear spin triples, but most of the nuclear spins would form either isolated spins or spin pairs whose pair-wise spin transitions are unlikely correlated[13,16].

We found the same numerical convergence behavior for the NV center in diamond. Therefore, we apply R_{bath} of 50 Å and r_{dipole} of 8 Å to all the divacancy and NV calculations.

Statistics for ensemble averages.

The quantum bath model described above suggests that the decoherence dynamics of a spin qubit coupled to a nuclear spin bath may significantly depend on the specific nuclear spin arrangement in a given bath, thus giving rise to variations in T_2 in an ensemble of random nuclear spin baths. Supplementary Figure 4 shows the histograms of T_2 (see Supplementary Figure 3 (b) for definition) of an (*kk*)-divacancy ensemble with 1000 different random nuclear spin baths at a static magnetic field of 115 G. T_2 shows significant variation across the nuclear samples, but eventually follows a normal distribution consistent with the central limit theorem. At a magnetic field of 115 G, the divancancy T_2 is centered around 1.3 ms, while some nuclear spin configurations give rise to 0.9 ms to 1.7 ms single spin coherence time.

To compare with experiments, we perform ensemble averages of the coherence functions and the T_2 times and we find that ensemble averages over 50 samples are good enough to produce numerically converged results. Supplementary Figure 4 (b) shows a direct comparison of the coherence function of the (*kk*)-divacancy spin ensemble averaged over 1000 samples to that averaged over 50 samples. We note that while the average over 1000 samples smooths out some minor noisy features on the coherence function, the overall shape is already well-converge with the average over 50 samples. Supplementary Table 1 summarizes ensemble T_2 and n of the (*kk*)-divacancy in 4*H*-SiC and the NV center in diamond at two magnetic fields of 65 G and 115 G, showing that the average over 50 samples provides converged T_2 and n for both systems.

Supplementary Note 3. Analytic equations of Hahn-echo coherence

Electron Spin Echo Envelop Modulation.

One of the main features in the coherence described in the main text is a rapid collapse and revival as a function of free evolution time t_{free}, which is known as electron spin echo envelop modulation (ESEEM) in the literature[14,17]. As hinted by the FFT power spectrum analysis shown in Figure 3 in the main article, ESEEM is driven by single nuclear spin precessions, hence the main ESEEM feature can be captured at the CCE-1 level of theory, i.e. independent nuclear spin approximation. As no nuclear-nuclear interactions are present in CCE-1, one can analytically solve the coherence equation by using, e.g. the product operator formalism[9] and the solution is given as:

$$\mathcal{L}_{\text{CCE1}}(t_{\text{free}}) = \prod_i (1 - 2k_i \sin^2\left(\sqrt{(\omega_i + A_i)^2 + B_i^2}\,\frac{t_{\text{free}}}{4}\right)\sin^2\left(\omega_i \frac{t_{\text{free}}}{4}\right), \quad (16)$$

where i runs over all single nuclear spins in the bath, ω_i is the nuclear Larmor frequency, and A_i and B_i (= $(B_{ix}^2+B_{iy}^2)^{1/2}$) are secular and pseudo-secular hyperfine interactions, respectively. k_i is called the modulation depth parameter[9], which is given as:

$$k_i = \frac{B_i^2}{(\omega_i + A_i)^2 + B_i^2}. \quad (17)$$

In Supplementary Figure 5, we calculate the coherence function at the three different magnetic fields from Figure 2 (b) in the main article by using the Supplementary Equation 16 and we note that the ESEEM oscillation is perfectly reproduced. When the applied static magnetic field increases the Larmor frequency increases, making the modulation depth parameter to go to zero. Therefore, the coherence oscillation amplitude is suppressed as observed in Supplementary Figure 5 and Figure 2 in the main article.

Pseudo-spin models of spin qubit decoherence.

To understand the decoherence dynamics of the (*kk*)-divacancy spin compared to the NV decoherence, we employ a pseudo-spin model, which has been applied to the NV center in the literature[18,19]. To check the applicability of the pseudo-spin model, we determine the most important Hamiltonian terms for the coherence decay. In Supplementary Figure 6, we calculate the coherence function of the divacancy in 4*H*-SiC and the NV center in diamond only with secular hyperfine interactions (A_i in Supplementary Equation 7) and compare it to the full CCE-2 calculation results. We note that the A_i-only calculations lacks the ESEEM feature (see Supplementary Equation 16 and 17 for the reason), while it captures the decay behavior especially for a magnetic field larger than 100 G for both NV and divacancy. For small magnetic fields under 100 G, there is significant contribution from the pseudo-secular hyperfine interactions as they

can effectively flip the nuclear spins owing to the small Zeeman splitting, inducing significant spin fluctuation in the bath. However, as the magnetic field increases more than B = 100 G, the Zeeman splitting increases and the pseudo-secular hyperfine induced nuclear spin flipping is suppressed, making the secular approximation for the hyperfine coupling good enough to describe the coherence decay.

Nuclear spins in diamond and 4H-SiC interact with each other by the nuclear dipole-dipole interaction (Supplementary Equation 6), inducing pairwise nuclear spin transitions. This can be easily seen by rewriting the dipolar Hamiltonian between nuclear spin n1 and n2 in Supplementary Equation 6 as follows[20]:

$$\mathcal{H}_{n1-n2} = \frac{\mu_0}{4\pi}\frac{\gamma_{n1}\gamma_{n2}\hbar^2}{r_{12}^3}(A + B + C + D + E + F), \quad (18)$$

where

$$\begin{aligned} A &= I_{1z}I_{2z}(1 - 3\cos^2\theta), \\ B &= -\frac{1}{4}(I_{1+}I_{2-} + I_{1-}I_{2+})(1 - 3\cos^2\theta), \\ C &= -\frac{3}{2}(I_{1+}I_{2z} + I_{1z}I_{2+})\sin\theta\cos\theta\, e^{-i\phi}, \\ D &= -\frac{3}{2}(I_{1-}I_{2z} + I_{1z}I_{2-})\sin\theta\cos\theta\, e^{+i\phi}, \\ E &= -\frac{3}{4}I_{1+}I_{2+}\sin^2\theta\, e^{-2i\phi}, \\ F &= -\frac{3}{4}I_{1-}I_{2-}\sin^2\theta\, e^{+2i\phi}. \end{aligned} \quad (19)$$

In Supplementary Figure 7, we calculate the Hahn-echo coherence of both divacancy and NV only with the AB, CD, or EF terms and compare them to the full Hahn-echo coherence function in order to identify the most important pairwise nuclear spin transitions. We find that at a magnetic field larger than 100 G, e.g. B = 300 G, CCE-2 calculations only keeping the nuclear spin flip-flop AB terms reproduce the full CCE-2 result, while similar calculations only using the CD or EF terms do not induce any coherence decay. For the NV center in diamond, the ↑↑ and ↓↓ configurations are well separated in energy from each other and from the ↑↓ and ↓↑ states due to the large Zeeman splitting, thus only the AB flip-flop transitions become the main pairwise transitions[18,19]. For the flip-flop transition, the ↑↓ and ↓↑ states are separated in energy by the difference in the hyperfine fields imposed by the electron spin shown in Supplementary Equation 7. For 4H-SiC, as explained in the main article, all possible pair-wise transitions for heterogeneous nuclear spin pairs are fully suppressed, thus only pairwise transitions in homogeneous spin pairs, e.g. either ^{13}C – ^{13}C or ^{29}Si – ^{29}Si, remain active for the coherence decay. Therefore, the same flip-flop AB terms in

the dipole-dipole coupling becomes the most important interaction channels even for the heterogeneous nuclear spin bath in 4*H*-SiC.

With the observations made so far (Supplementary Figure 6 and Supplementary Figure 7), we can construct a pseudo-spin model for a homogeneous nuclear spin pair interacting with a spin qubit by keeping only the secular hyperfine term (A_i) and the flip-flop term from the dipole-dipole interaction. The Hilbert space for the pseudo-spin model only contains the two ↑↓ and ↑↓ nuclear spin states and the pseudo-spin Hamiltonian (for nuclear spin 1 and 2) can be written as:

$$\mathcal{H}_{12}^{m_s} = D_{12}^{m_s} J_x + \Omega_{12}^{m_s} J_z, \tag{20}$$

where m_s is the electron spin sub-level (either 0 or 1 for NV and divacancy), and J_z and J_x are fictious spin-1/2 operators. $\Omega_{12}^{m_s}$ is a pseudo-spin frequency depending on the electron spin sublevel and in our case, it is given as:

$$\begin{gathered} \Omega_{12}^{m_s=+1} = \Delta A_{12} = A_1 - A_2, \\ \Omega_{12}^{m_s=0} = 0. \end{gathered} \tag{21}$$

$D_{12}^{m_s}$ is a pseudo-spin transition rate conditioned on the electron spin state, derived from the secular nuclear dipole-dipole interaction:

$$D_{12}^{m_s=+1} = D_{12}^{m_s=0} \equiv D_{12} = \frac{1}{2}\left(\frac{\mu_0}{4\pi}\frac{\gamma_1\gamma_2\hbar^2}{r_{12}^3}\right)(3\cos^2\theta_{12} - 1). \tag{22}$$

Then, the Hahn-echo coherence function of the divacancy spin (or the NV center) coupled to this homogeneous nuclear spin pair is given as:

$$\mathcal{L}_{\text{pair}}(t_{\text{free}}) = 1 - K_{12}\sin^2\left(\sqrt{(\Delta A_{12})^2 + D_{12}^2}\,\frac{t_{\text{free}}}{4}\right)\sin^2\left(D_{12}\frac{t_{\text{free}}}{4}\right), \tag{23}$$

where

$$K_{12} = \frac{(\Delta A_{12})^2}{(\Delta A_{12})^2 + D_{12}^2}. \tag{24}$$

Supplementary Figure 8 compares the coherence function calculated using the pseudo-spin model to the coherence decay from the full CCE calculation for both the NV and divacancy defects. We observe that the pseudo-spin model reproduces the overall coherence decay well for both NV and divacancy.

Supplementary References

1. Breuer, H. P. & Petruccione, F. *The Theory of Open Quantum Systems*. (OUP Oxford, 2007).
2. Balasubramanian, G., Neumann, P., Twitchen, D., Markham, M., Kolesov, R., Mizuochi, N., Isoya, J., Achard, J., Beck, J., Tissler, J., Jacques, V., Hemmer, P. R., Jelezko, F. & Wrachtrup, J. Ultralong spin coherence time in isotopically engineered diamond. *Nat. Mater.* **8,** 383–387 (2009).
3. Stanwix, P. L., Pham, L. M., Maze, J. R., Le Sage, D., Yeung, T. K., Cappellaro, P., Hemmer, P. R., Yacoby, A., Lukin, M. D. & Walsworth, R. L. Coherence of nitrogen-vacancy electronic spin ensembles in diamond. *Phys. Rev. B* **82,** 201201(R) (2010).
4. Falk, A. L., Buckley, B. B., Calusine, G., Koehl, W. F., Dobrovitski, V. V., Politi, A., Zorman, C. A., Feng, P. X. L. & Awschalom, D. D. Polytype control of spin qubits in silicon carbide. *Nature Comm.* **4,** 1819 (2013).
5. Christle, D. J., Falk, A. L., Andrich, P., Klimov, P. V., Hassan, J. U., Son, N. T., Janzen, E., Ohshima, T. & Awschalom, D. D. Isolated electron spins in silicon carbide with millisecond coherence times. *Nat. Mater.* **14,** 160–163 (2014).
6. Klauder, J. R. & Anderson, P. W. Spectral Diffusion Decay in Spin Resonance Experiments. *Phys. Rev.* **125,** 912–932 (1962).
7. Hartman, J. S., Berno, B., Hazendonk, P., Kirby, C. W., Ye, E., Zwanziger, J. & Bain, A. D. NMR Studies of Nitrogen Doping in the 4H Polytype of Silicon Carbide: Site Assignments and Spin−Lattice Relaxation. *J. Phys. Chem. C* **113,** 15024–15036 (2009).
8. Hartman, J. S., Narayanan, A. & Wang, Y. Spin-Lattice Relaxation in the 6H Polytype of Silicon Carbide. *J. Am. Chem. Soc.* **116,** 4019–4027 (1994).
9. Schweiger, A. & Jeschke, G. *Principles of Pulse Electron Paramagnetic Resonance*. (Oxford University Press, 2001).
10. Jarmola, A., Acosta, V. M., Jensen, K., Chemerisov, S. & Budker, D. Temperature- and Magnetic-Field-Dependent Longitudinal Spin Relaxation in Nitrogen-Vacancy Ensembles in Diamond. *Phys. Rev. Lett.* **108,** 197601 (2012).
11. Falk, A. L., Klimov, P. V., Buckley, B. B., Ivády, V., Abrikosov, I. A., Calusine, G., Koehl, W. F., Gali, A. & Awschalom, D. D. Electrically and Mechanically Tunable Electron Spins in Silicon Carbide Color Centers. *Phys. Rev. Lett.* **112,** 187601 (2014).
12. Yang, W. & Liu, R.-B. Quantum many-body theory of qubit decoherence in a finite-size spin bath. *Phys. Rev. B* **78,** 085315 (2008).
13. Witzel, W. M., de Sousa, R. & Das Sarma, S. Quantum theory of spectral-diffusion-induced electron spin decoherence. *Phys. Rev. B* **72,** 161306(R) (2005).
14. Van Oort, E. & Glasbeek, M. Optically detected low field electron spin echo envelope modulations of fluorescent N-V centers in diamond. *Chemical Physics* **143,** 131–140 (1990).
15. Guennebaud, G., Jacob, B. & others. *Eigen v3*. (http://eigen.tuxfamily.org, 2010).
16. Yao, W., Liu, R.-B. & Sham, L. J. Theory of electron spin decoherence by interacting nuclear spins in a quantum dot. *Phys. Rev. B* **74,** 195301 (2006).
17. Mims, W. B. Envelope Modulation in Spin-Echo Experiments. *Phys. Rev. B* **5,** 2409–2419 (1972).
18. Maze, J. R., Taylor, J. M. & Lukin, M. D. Electron spin decoherence of single nitrogen-vacancy defects in diamond. *Phys. Rev. B* **78,** 094303 (2008).
19. Zhao, N., Ho, S.-W. & Liu, R.-B. Decoherence and dynamical decoupling control of nitrogen vacancy center electron spins in nuclear spin baths. *Phys. Rev. B* **85**, 115303 (2012).
20. Slichter, C. P. *Principles of Magnetic Resonance*. (Springer Berlin Heidelberg, 1996).
